# Enhancing effects of diphenyl diselenide and β-hydroxy β-methylbutyrate combined with exercise on neuroprotection, memory, mitochondrial function, muscle function, and inflammation regulation in older adults and age-related diseases

**DOI:** 10.3389/fnut.2026.1770666

**Published:** 2026-06-18

**Authors:** Ranran Huang, Wendan Pang

**Affiliations:** 1 Department of Geriatric, The Third Affiliated Hospital of Zhejiang Chinese Medical University, Hangzhou, Zhejiang, China; 2 Department of Rehabilitation, Tiantai People’s Hospital of Zhejiang Province (Tiantai Branch of Zhejiang Provincial People’s Hospital), Taizhou, Zhejiang, China

**Keywords:** cognitive function, diphenyl diselenide, exercise, physical outcomes, β-hydroxy β-methylbutyrate

## Abstract

Aging and age-associated pathologies are characterized by a gradual deterioration in cognitive abilities, neuroprotective mechanisms, mitochondrial efficacy, skeletal muscle mass, and overall functional capacity, frequently aggravated by oxidative stress and persistent inflammation. Physical exercise is extensively acknowledged for its role in ameliorating these age-related detriments; however, its effectiveness may be further enhanced through specific nutritional supplementation. Diphenyl diselenide (PhSe₂) represents a selenium-derived compound endowed with significant antioxidant and anti-inflammatory capabilities, whereas β-hydroxy β-methylbutyrate (HMB) serves as a metabolite of leucine that is recognized for its ability to augment muscle mass, strength, and protein synthesis. This review consolidates contemporary findings regarding the synergistic effects of PhSe₂ and HMB supplementation, in conjunction with exercise interventions, within aging models and age-related disorders. The amalgamation of these compounds with organized physical activity seemingly intensifies neuroprotective mechanisms, enhances cognitive performance, optimizes mitochondrial functionality, modulates inflammatory cytokines, and sustains skeletal muscle mass and functional capabilities. Mechanistically, these advantages are facilitated through the modulation of oxidative stress pathways, mitochondrial biogenesis, anti-apoptotic signaling, and the turnover of muscle proteins. Collectively, PhSe₂ and HMB, in conjunction with exercise, constitute a promising multi-targeted approach aimed at mitigating age-related physiological decline and promoting healthy aging. Additional preclinical and clinical investigations are necessary to optimize dosage, timing, and long-term efficacy within elderly populations and individuals suffering from age-associated disorders.

## 1 Introduction

Aging represents a multifaceted, systemic phenomenon distinguished by the gradual erosion of physiological integrity, which ultimately results in functional deterioration across neural, muscular, and immune systems. Integral to this decline are interrelated mechanisms such as neurodegeneration, mitochondrial impairment, sarcopenia, and persistent low-grade inflammation (collectively referred to as “inflammaging”) (1). These mechanisms intensify neuronal susceptibility, diminish synaptic plasticity, hinder mitochondrial bioenergetics, and facilitate muscle wasting, thus contributing to the emergence and advancement of age-associated pathologies, including Alzheimer’s disease (AD), Parkinson’s disease (PD), frailty syndromes, and metabolic disorders (2–4). An expanding body of evidence suggests that disrupted redox homeostasis and compromised cellular stress responses are fundamental to numerous pathological alterations, serving as precursors to both neurocognitive decline and muscular dysfunction (5, 6).

Physical exercise has been extensively acknowledged as one of the most potent non-pharmacological interventions to mitigate age-related deficits. Its well-documented influence on neurotrophic signaling, mitochondrial biogenesis, metabolic coordination, and immune modulation renders it a formidable modulator of healthy aging (7, 8). Nevertheless, despite its extensive advantages, there exists considerable variability in the degree of responsiveness to exercise among individuals, particularly within older adults exhibiting reduced cellular resilience. This heterogeneity highlights the necessity for the incorporation of nutraceuticals that possess the potential to enhance the biological impacts of exercise. Among such candidates, diphenyl diselenide (PhSe₂) and β-hydroxy β-methylbutyrate (HMB) have garnered increasing scholarly attention. PhSe₂ is a selenium-derived compound recognized for its redox-modulatory, antioxidant, anti-inflammatory, and neuroprotective properties, exerting its effects through glutathione-dependent pathways and the preservation of mitochondrial function (9). HMB, a metabolite derived from leucine, promotes muscle protein turnover, improves mitochondrial efficacy, modulates inflammatory responses, and has shown emergent roles in cognitive functions and neuroprotective mechanisms (10–12).

While the distinct effects of PhSe₂, HMB, and exercise have been extensively documented in the literature, no prior review has amalgamated their collective or potentially synergistic effects on neuroprotection, memory enhancement, mitochondrial functionality, muscular performance, and inflammatory regulation within the framework of aging and age-associated pathologies. Existing reviews predominantly analyze these agents in isolation, frequently within narrowly delineated physiological contexts. Such reductionist methodologies hinder a comprehensive understanding of how multimodal interventions may interact to yield compounded benefits across systems that are intrinsically interconnected within the aging phenotype. A thorough examination of the literature further indicates considerable fragmentation: studies exhibit considerable variability in model organisms, dosages, exercise modalities, and assessed endpoints, thereby constraining the capacity to generalize findings or formulate integrative therapeutic frameworks. The lack of a holistic, mechanistically informed synthesis that encompasses neural, muscular, metabolic, and inflammatory dimensions signifies a substantial void that the current review aspires to address.

Several pivotal insights arise from the synthesis of accumulated empirical data and experiential knowledge within the domain. The process of aging is fundamentally influenced by a variety of biological perturbations, necessitating interventions that concurrently target multiple pathways. Physical activity remains the cornerstone of strategies aimed at promoting healthy aging; however, its efficacy may be significantly augmented by the incorporation of nutraceuticals that operate through complementary biological mechanisms. PhSe₂ and HMB present unique yet synergistically harmonious benefits— PhSe₂ via modulation of redox states and mitochondrial function, and HMB through the regulation of muscle metabolism, systemic inflammation, and potential enhancement of neurocognitive functions (9, 13–15). The amalgamation of these compounds with exercise exhibits considerable potential for yielding superior outcomes in comparison to any singular intervention, especially for the elderly population experiencing accelerated declines in neuromuscular and cognitive faculties. Looking ahead, various prospective avenues are critical to propel advancements within this domain. It is imperative to conduct synergy-focused clinical trials that integrate PhSe₂, HMB, and systematically structured exercise regimens, ideally employing harmonized dosing methodologies and stringent functional endpoints. Future investigations ought to increasingly embrace biomarker-driven and individualized strategies, utilizing mitochondrial assays, redox profiles, inflammatory markers, and neurocognitive evaluations to customize interventions according to distinct physiological requirements. The implementation of multi-omics and systems biology frameworks will be pivotal for elucidating how synergistic interventions influence the interrelated pathways of aging. Ultimately, the amalgamation of PhSe₂ and HMB with exercise regimens may provide the foundation for next-generation precision approaches aimed at enhancing resilience, maintaining functional capacity, and alleviating the complex decline associated with the aging process.

## 2 Diphenyl diselenide (PhSe₂) and aging

### 2.1 Chemical properties and safety profile

PhSe₂ represents an organoselenium compound, characterized by a central selenium-selenium bond, which is adjacently positioned between two phenyl rings. This molecular architecture imparts significant lipophilicity, facilitating effective permeation across cellular membranes and interaction with intracellular redox systems. PhSe₂ readily engages in thiol-disulfide exchange reactions, thereby enabling it to simulate the activity of glutathione peroxidase and influence oxidative equilibrium (16). In comparison to alternative selenium compounds, PhSe₂ demonstrates a commendable safety profile, marked by minimal acute toxicity and a wide therapeutic index when administered at physiologically relevant dosages (9, 17). Its chemical robustness and predictable redox behavior mitigate the potential for adverse pro-oxidant effects, positioning it as a promising candidate for therapeutic interventions related to aging.

### 2.2 Neuroprotective effects of PhSe₂

The neuroprotective characteristics of PhSe₂ assume particular importance within the framework of senescence, a period during which neurons exhibit heightened vulnerability to oxidative stress, excitotoxicity, and inflammatory insult. Empirical evidence derived from experimental models illustrates that PhSe₂ sustains neuronal viability through the regulation of glutathione metabolism, modulation of the thioredoxin system, inhibition of reactive oxygen species (ROS) production, and preservation of synaptic structural integrity (18, 19). These mechanisms collectively contribute to the mitigation of neuronal loss observed in age-associated neurodegenerative conditions such as AD and PD. By attenuating excitotoxic and apoptotic signaling pathways, PhSe₂ promotes neuronal survival amidst the cellular stressors prevalent in aging tissues.

### 2.3 Antioxidant and anti-inflammatory mechanisms

PhSe₂ demonstrates significant antioxidant properties by diminishing lipid peroxidation, preserving glutathione concentrations, and regulating essential antioxidant enzymes, including catalase and superoxide dismutase. These physiological processes contribute to the re-establishment of redox homeostasis, which progressively deteriorates with advancing age (9, 20). Beyond its antioxidant capabilities, PhSe₂ also manifests anti-inflammatory effects through the attenuation of pro-inflammatory cytokines such as TNF-α and IL-1β, as well as the inhibition of NF-κB signaling pathways (21). This synergistic interplay of antioxidant and anti-inflammatory pathways is particularly pertinent in addressing “inflammaging,” a persistent low-grade inflammatory condition that hastens functional deterioration across various organ systems.

### 2.4 Impact on memory and cognitive function

Emerging research indicates that PhSe₂ enhances both memory and cognitive performance through its neuroprotective, antioxidant, and anti-inflammatory properties. In murine models, the administration of PhSe₂ has been correlated with enhancements in learning capacity, spatial memory, and recognition memory—effects that are ascribed to the compound’s capability to maintain hippocampal redox equilibrium, attenuate neuroinflammation, and facilitate synaptic plasticity (22). By safeguarding synaptic proteins and modulating glutamatergic neurotransmission, PhSe₂ may play a role in alleviating the progressive cognitive deterioration associated with the aging process.

### 2.5 Effects on mitochondria and cellular energy metabolism

Mitochondrial dysfunction represents a fundamental characteristic of the aging process and is implicated in the disruption of energy metabolism, increased production of ROS, and the activation of apoptotic signaling pathways. PhSe₂ has been shown to possess the capacity to stabilize the integrity of mitochondrial membranes, enhance the efficiency of the electron transport chain, diminish the formation of mitochondrial ROS, and inhibit mitochondrial permeability transition (19). These mechanisms are instrumental in preserving ATP synthesis and augmenting the overall efficiency of cellular energy metabolism. By reinforcing mitochondrial resilience, PhSe₂ may serve to mitigate age-associated declines in the functional capacities of both neuronal and muscle cells.

## 3 β-hydroxy β-methylbutyrate (HMB) and aging

### 3.1 Metabolic role of HMB in muscle physiology

HMB is a bioactive derivative of the indispensable branched-chain amino acid leucine. It plays a pivotal role in the preservation of muscle cell integrity and the maintenance of metabolic equilibrium. HMB exerts its influence on muscle physiology predominantly by enhancing membrane stability, augmenting mitochondrial function, and facilitating anabolic signaling via pathways including mTOR, AMPK, and PI3K/Akt (10, 23). Moreover, HMB is implicated in the synthesis of cholesterol within muscle tissue, a critical process for the repair of the sarcolemma. This array of metabolic activities assumes increased significance as individuals age, a period characterized by heightened oxidative stress, compromised regenerative capacity, and diminished efficiency in protein turnover.

### 3.2 Effects on muscle mass, strength, and function

The reduction in muscle mass, strength, and physical functionality constitutes a significant characteristic of the aging process and is frequently linked to frailty and diminished quality of life. HMB has surfaced as a potentially beneficial supplement for alleviating age-associated muscle deterioration. Both clinical and preclinical investigations suggest that the supplementation of HMB contributes to the preservation or augmentation of lean muscle mass, enhances muscle strength, and improves functional performance in elderly individuals and those suffering from muscle wasting (12, 24). These beneficial outcomes are ascribed to the promotion of protein synthesis, the attenuation of protein degradation, and the enhancement of muscle cell viability under conditions of stress. By bolstering neuromuscular integrity, HMB may serve to postpone or mitigate the advancement of sarcopenia.

### 3.3 Influence on protein synthesis and degradation

HMB exerts significant regulatory influences on the metabolic processes of skeletal muscle protein. On the anabolic front, HMB activates the mTOR signaling cascade, which results in enhanced protein synthesis and augmented muscle fiber hypertrophy (25). Additionally, HMB upregulates the expression of IGF-1, an essential growth factor that plays a critical role in muscle repair and regeneration. Conversely, on the catabolic front, HMB inhibits proteolytic pathways, including the ubiquitin–proteasome system and caspase-dependent proteolysis, both of which are elevated during aging, immobilization, and metabolic stress (26, 27). Through this dual mechanism—promoting synthesis while simultaneously suppressing degradation—HMB plays a pivotal role in the preservation of muscle mass and the maintenance of muscle quality within aging populations.

### 3.4 Anti-inflammatory and oxidative stress modulation

Chronic low-grade inflammation and heightened oxidative stress constitute significant factors contributing to the decline of muscular function associated with aging. HMB has been shown to possess the capability to diminish inflammatory signaling pathways through the reduction of pro-inflammatory cytokines such as TNF-α, IL-6, and CRP, alongside the downregulation of NF-κB activity (25). IL-6 is a pleiotropic cytokine that functions not only as a pro-inflammatory mediator but also as a myokine and adipokine involved in the regulation of insulin sensitivity, energy metabolism, and adipocyte browning. In the context of exercise, IL-6 may exert beneficial metabolic and anti-inflammatory effects, highlighting its dual and context-dependent role.

Furthermore, HMB fortifies antioxidant defenses by influencing glutathione levels, decreasing the production of ROS, and enhancing mitochondrial redox equilibrium (28). The collective impact of these effects serves to alleviate oxidative damage to muscle fibers, promote muscle recovery, and counteract the catabolic milieu commonly observed in aging tissues (28). Through the dual mitigation of inflammation and oxidative stress, HMB fosters a more advantageous biochemical environment conducive to the maintenance of muscle functionality.

### 3.5 Potential benefits for neuroprotection and mitochondrial function

While traditionally examined for its influence on skeletal muscle physiology, an increasing body of evidence suggests that HMB may also have neuroprotective properties. HMB promotes neuronal integrity by facilitating mitochondrial biogenesis, reinforcing mitochondrial membrane stability, and mitigating neuroinflammation that are particularly pertinent to cognitive deterioration and neurodegenerative disorders linked to the aging process (23, 29). Research has demonstrated that HMB activates signaling cascades associated with cellular survival, diminishes excitotoxic stress, and modulates neuronal energy metabolism (14). Moreover, enhancements in mitochondrial efficiency may lead to improved ATP availability in both muscular and neural tissues. These observations indicate that HMB possesses potential therapeutic significance not only for the preservation of muscle mass but also for the enhancement of cognitive resilience and neuronal functionality in aging demographics.

Recent studies in rodent models have demonstrated that HMB supplementation can modulate central nervous system function by enhancing mitochondrial bioenergetics, improving neuronal resilience, and attenuating neuroinflammatory processes. Mechanistically, HMB has been shown to promote mitochondrial biogenesis and stabilize mitochondrial membrane potential, thereby preserving ATP production and reducing the accumulation of reactive oxygen species (15). In addition, HMB appears to influence key signaling pathways involved in neuronal survival, including activation of the PI3K/Akt and MAPK/ERK cascades, which are critical for synaptic plasticity and cell survival. Emerging evidence further suggests that HMB may regulate neuroinflammation by suppressing microglial activation and reducing the expression of pro-inflammatory cytokines, thereby contributing to a more favorable neural microenvironment. Notably, recent findings (15) indicate that HMB can influence brain energy metabolism and neurotransmission, potentially through modulation of metabolic intermediates and enhancement of neuronal metabolic flexibility. These effects may be particularly relevant in the context of aging and neurodegenerative diseases, where impaired energy metabolism and chronic inflammation are central pathological features. Collectively, these data support the concept that HMB is not solely a muscle-targeted metabolite but also a promising neuromodulatory compound with potential therapeutic relevance in preserving cognitive function and mitigating neurodegenerative processes.

A recent study provided compelling evidence supporting the neuroprotective role of HMB in rodent models (30). The authors demonstrated that HMB supplementation significantly improved cognitive performance and neuronal function, particularly in aging and metabolically compromised animals. Mechanistically, HMB was shown to enhance brain energy metabolism by modulating key metabolic pathways involved in mitochondrial respiration and ATP production. In addition, the study reported a reduction in neuroinflammatory markers, including decreased microglial activation and lower expression of pro-inflammatory cytokines, suggesting an anti-inflammatory effect within the central nervous system. Importantly, HMB treatment was associated with improved synaptic integrity and plasticity, potentially through activation of pro-survival signaling pathways such as PI3K/Akt. Recent experimental evidence in AD models further supports the neurobiological effects of HMB. Specifically, HMB supplementation has been shown to enhance synaptic plasticity and cognitive performance in transgenic AD mice, accompanied by increased expression of synaptic proteins such as SNAP25 and PSD-95, as well as activation of CREB signaling pathways (31). These findings highlight HMB as a metabolically active compound capable of influencing brain function and resilience, thereby supporting its potential as a therapeutic candidate for neuroprotection in aging and neurodegenerative conditions (30).

## 4 Exercise as a synergistic strategy

### 4.1 Mechanisms of exercise-induced neuroprotection and muscle preservation

Physical activity functions as a multifaceted biological stimulus capable of activating intricate signaling pathways that safeguard both the neural and muscular systems throughout the aging process. Engagement in aerobic and resistance training mitigates age-related neuronal susceptibility by augmenting synaptic plasticity, fostering neurogenesis, and enhancing cerebral perfusion (32). Furthermore, exercise sustains neuromuscular integrity through the stimulation of satellite cell proliferation, the attenuation of proteolytic activity, and the preservation of neuromuscular junction stability that are essential mechanisms that combat sarcopenia and functional deterioration (33). At the cellular level, physical activity initiates adaptive stress responses that curtail oxidative damage, bolster DNA repair processes, and uphold redox homeostasis, thus contributing to enduring neuroprotection and muscle conservation within aging demographics (34, 35).

In the context of neurodegenerative disorders, physical exercise has emerged as a potent non-pharmacological intervention capable of modulating multiple disease-relevant pathways. Recent evidence from experimental models of AD and PD indicates that exercise exerts neuroprotective effects by attenuating neuroinflammation, improving mitochondrial function, and enhancing synaptic plasticity. For instance, findings from a study (36) demonstrated that exercise can regulate brain metabolic and signaling pathways associated with neuronal survival, including improvements in mitochondrial efficiency and reductions in oxidative stress. Similarly, another study (37) highlighted the role of exercise in modulating neuroimmune interactions, showing that physical activity reduces microglial activation and shifts inflammatory signaling toward an anti-inflammatory profile. These immunomodulatory effects are particularly relevant in neurodegenerative diseases, where chronic neuroinflammation contributes to neuronal dysfunction and disease progression. Furthermore, exercise-induced upregulation of neurotrophic factors such as BDNF supports synaptic remodeling and cognitive resilience. Collectively, these findings reinforce the concept that exercise acts through integrated metabolic, inflammatory, and neurotrophic mechanisms, making it a critical component in strategies aimed at preventing or slowing the progression of neurodegenerative disorders.

### 4.2 Effects on BDNF, inflammatory cytokines, and mitochondrial biogenesis

One of the most precisely characterized mediators of the neuroprotective consequences of physical exercise is the upregulation of brain-derived neurotrophic factor (BDNF), which serves as a critical regulator of neuronal viability, dendritic development, and cognitive processing. Both acute and chronic physical exercise significantly elevate peripheral and central BDNF concentrations, resulting in augmented hippocampal plasticity and cognitive resilience (38). Furthermore, physical activity mitigates chronic low-grade inflammation through the reduction of circulating pro-inflammatory cytokines such as TNF-α, IL-6, and IL-1β, while concurrently enhancing the levels of anti-inflammatory myokines including IL-10 and irisin (39). These immunomodulatory responses are intricately associated with the enhancement of mitochondrial health. Physical exercise stimulates PGC-1α, AMPK, and NRF-1, which culminates in increased mitochondrial biogenesis, augmented respiratory efficiency, and improved antioxidant defenses—adaptations that effectively counteract mitochondrial dysfunction, a defining characteristic of aging and neurodegeneration (40).

### 4.3 Exercise-mediated improvements in cognitive and physical performance

The structural and molecular advantages associated with physical exercise manifest as considerable enhancements in cognitive functioning among the elderly population. Consistent engagement in physical activities promotes improvements in executive function, memory consolidation, processing speed, and attentional capacity, with aerobic training exhibiting particularly pronounced effects on the volume and connectivity of the hippocampus (41). From a functional perspective, exercise augments muscle mass, power, and endurance, while concurrently enhancing gait velocity, balance, and overall physical performance including elements that mitigate the risk of disability and improve the quality of life in aging demographics (42). Notably, the cognitive and physical advancements seem to be synergistic, as enhancements in muscular function and metabolic health facilitate improved brain perfusion, decreased inflammation, and increased resilience against neurodegenerative processes (43). These cumulative effects provide a compelling justification for the integration of exercise with nutraceutical strategies such as PhSe₂ and HMB to intensify neuroprotective, mitochondrial, anti-inflammatory, and functional benefits throughout the aging process.

## 5 Combined effects of PhSe₂, HMB, and exercise

### 5.1 Synergistic impacts on neuroprotection and memory

The concurrent administration of PhSe₂ and HMB, in conjunction with structured physical exercise, is mechanistically situated to elicit synergistic neuroprotective effects that surpass the advantages of each individual intervention. PhSe₂ demonstrates pronounced antioxidant, anti-inflammatory, and thiol-redox modulating capabilities, thereby safeguarding neurons from oxidative and excitotoxic injuries (44), whereas HMB contributes to cellular integrity by fortifying neuronal membranes, augmenting protein synthesis, and mitigating catabolic processes (23). Physical exercise augments these mechanisms by fostering neurogenesis, enhancing synaptic plasticity, and facilitating BDNF-mediated cognitive improvements (41). Collectively, these effects may culminate in significantly enhanced learning and memory capabilities within aging populations, partially through the convergent modulation of hippocampal signaling pathways, maintenance of mitochondrial homeostasis, and attenuation of neuroinflammatory processes.

### 5.2 Modulation of mitochondrial function and oxidative stress

Mitochondrial dysfunction and oxidative stress are pivotal characteristics of the aging process and age-associated neurodegenerative diseases. PhSe₂ enhances mitochondrial efficiency through the augmentation of glutathione-dependent redox cycling and the safeguarding of respiratory complexes against oxidative damage (45). HMB additionally promotes mitochondrial integrity by stimulating PGC-1α, facilitating ATP synthesis, and diminishing mitochondrial proteolysis (46). Exercise serves as a third synergistic element by promoting mitochondrial biogenesis, enhancing oxidative phosphorylation, and fortifying intrinsic antioxidant mechanisms (40). When these interventions are combined, they may yield either additive or synergistic enhancements in mitochondrial membrane potential, ROS scavenging, and metabolic resilience—ultimately decelerating cellular aging and fostering neuroenergetic efficiency.

### 5.3 Enhancement of muscle mass, strength, and physical function

HMB is widely recognized for its role in promoting muscle protein synthesis, mitigating ubiquitin–proteasome–mediated degradation, and optimizing overall nitrogen balance within the body (47). PhSe₂, although predominantly investigated within neurological contexts, plays an indirect role in muscle preservation by attenuating systemic oxidative stress and sustaining cellular redox equilibrium (44). Physical exercise amplifies these molecular mechanisms through mechanical loading, activation of satellite cells, and enhancement of anabolic signaling cascades such as mTOR and IGF-1 (48). The synergistic effect of HMB and exercise has already been recognized as an effective defense against sarcopenia; the inclusion of PhSe₂ may further enhance functional outcomes by reducing oxidative damage to muscle mitochondria and contractile proteins. Consequently, this triadic combination may yield superior enhancements in muscle mass, strength, endurance, and overall physical functionality in the aging population.

### 5.4 Integrated regulation of inflammatory pathways

Chronic low-grade inflammation serves to expedite neurodegenerative processes, muscle atrophy, and metabolic deterioration throughout the aging process. PhSe₂ demonstrates significant anti-inflammatory capabilities by attenuating NF-κB signaling pathways and diminishing pro-inflammatory mediators such as TNF-α and IL-1β (9). HMB additionally mitigates inflammation by curtailing myofiber damage, inhibiting the production of proteolysis-related cytokines, and elevating anti-inflammatory biomarkers (23). Physical exercise influences systemic inflammation by reducing cytokines derived from visceral adipose tissue, augmenting IL-10 levels, inducing myokines with anti-inflammatory properties, and strengthening immune regulatory mechanisms (49). These therapeutic approaches converge in their targeting of inflammatory pathways, indicating a synergistic anti-inflammatory impact that may facilitate the restoration of homeostatic equilibrium, safeguard neural and muscular structures, and diminish the advancement of age-associated pathologies.

## 6 Clinical and preclinical evidence of amplifying effects of PhSe₂ combined with exercise in aging and age-related diseases

**Figure 1:**
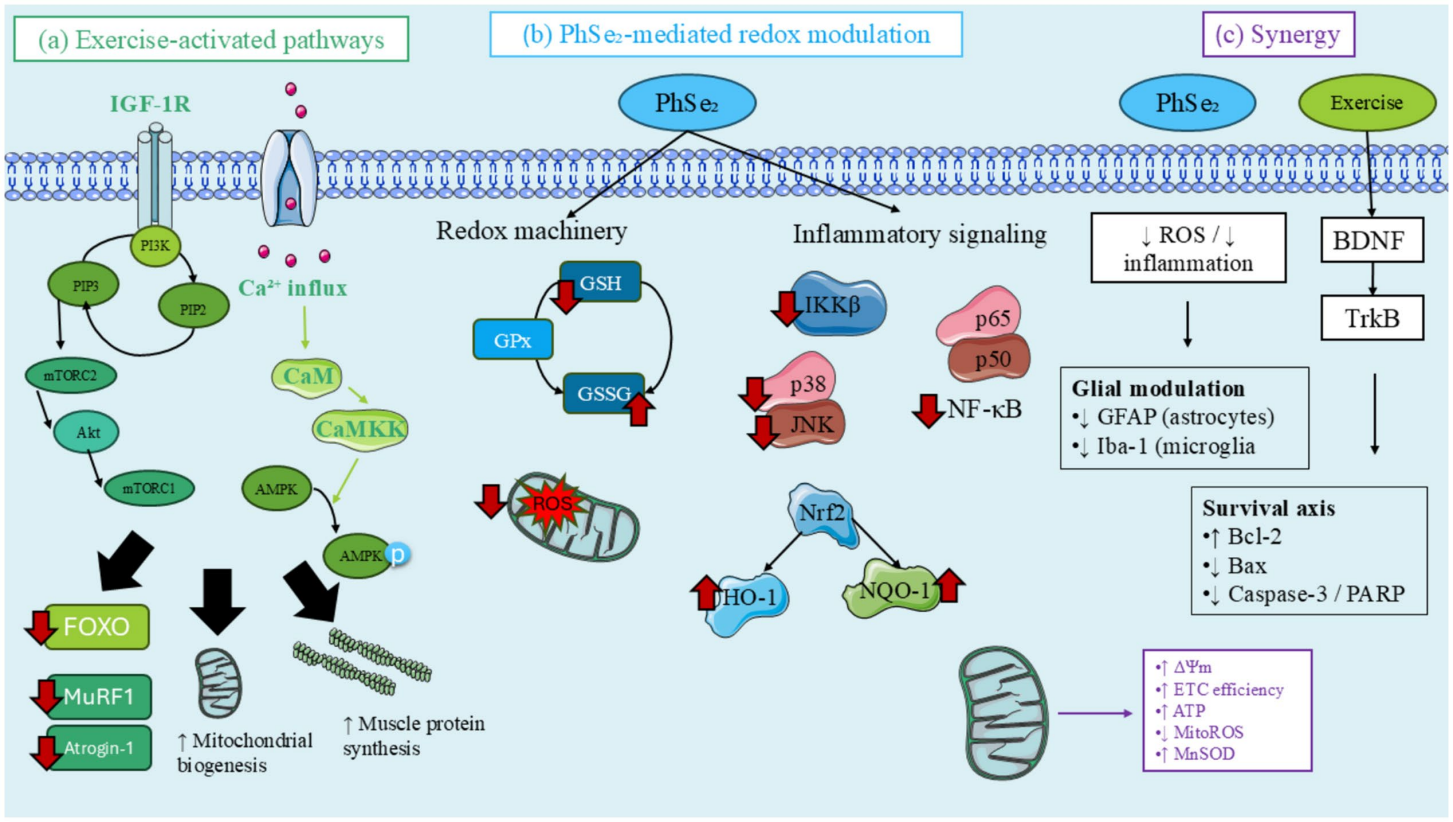
Amplifying molecular mechanisms underlying the combined effects of diphenyl diselenide (PhSe_2_) and exercise in aging and age-related diseases. The schematic illustrates the integrated and convergent signaling pathways through which physical exercise and PhSe_2_ supplementation modulate aging-associated neurodegenerative, metabolic, and inflammatory processes, based predominantly on evidence from preclinical aging models. **(a)** Exercise activates mechanosensitive and metabolic signaling cascades, including Ca^2+^ influx, AMPK, CaMKII, and IGF-1/PI3K/Akt/mTOR pathways, leading to enhanced mitochondrial biogenesis via PGC-1*α*, improved muscle protein synthesis, and suppression of FOXO-mediated atrophy genes (e.g., MuRF1 and Atrogin-1). **(b)** PhSe_2_ exerts redox-modulatory and anti-inflammatory effects by mimicking glutathione peroxidase activity, improving the GSH/GSSG ratio, reducing cytosolic and mitochondrial reactive oxygen species (ROS), activating Nrf2-dependent antioxidant defenses (HO-1, NQO1), and attenuating pro-inflammatory signaling pathways including IKK*β*/NF-κB and stress-activated MAPKs (JNK, p38). **(c)** Convergence of exercise- and PhSe_2_-induced signaling in the aging brain enhances neuroprotection, characterized by increased BDNF/TrkB signaling, activation of cAMP/PKA/CREB pathways, upregulation of anti-apoptotic proteins (Bcl-2), suppression of pro-apoptotic mediators (Bax, caspase-3, PARP cleavage), and reduced astrocytic and microglial activation (GFAP, Iba-1), collectively supporting synaptic plasticity, GABAergic neurotransmission, and cognitive function.

Aging encompasses neurochemical modifications that influence structural proteins and neurotransmitter systems. Physical exercise is recognized for its capacity to improve overall health, whereas PhSe₂ demonstrates significant antioxidant, anti-inflammatory, and neuroprotective effects in rodent models. The integration of pharmacological and non-pharmacological strategies has been suggested as a means to mitigate age-associated alterations. An investigation examined the synergistic impact of aquatic exercise and dietary supplementation of (PhSe)₂ on [^3^H]GABA uptake in aged rat models. Male Wistar rats, aged 24 months, were administered a diet enriched with 1 ppm (PhSe)₂ and participated in daily 20-min swimming sessions over a duration of 4 weeks. Post-treatment, [^3^H]GABA uptake was assessed in both the cerebral cortex and the striatum. The results indicated that the combination of (PhSe)₂ and exercise effectively countered age-related declines in GABA uptake, suggesting a favorable modulation of GABAergic activity within these cerebral regions (50) (Table 1 and Figure 1). Aging is associated with a progressive decline in cognitive functions such as learning and memory. Another work aimed to elucidate the effects of these interventions on hippocampal proteins that are associated with neuroprotective mechanisms. Male Wistar rats, aged 27 months, were administered a diet containing 1 ppm PhSe₂ while engaging in daily swimming activities with a modest workload for a duration of 4 weeks. Subsequent analysis of hippocampal tissue was conducted utilizing techniques such as western blotting and immunohistochemistry. The synergistic effect of the interventions resulted in an elevation of proteins implicated in neuroprotection, concurrently diminishing the expression of apoptotic and neuroinflammatory markers. Collectively, these findings demonstrated that the combination of physical exercise and dietary PhSe₂ serves to augment hippocampal neuroprotection in aged rats, thereby mitigating the underlying mechanisms responsible for cognitive deterioration (51). The aim of study was to explore the effects of an integrated regimen of aquatic exercise alongside a dietary incorporation of 1 ppm PhSe₂ on age-related metabolic dysfunctions within the hepatic tissue of senescent rats. Elderly male Wistar rats exhibited an increase in epididymal adipose tissue, compromised hepatic enzymatic functionality pertinent to glucose metabolism, elevated triglyceride concentrations, and heightened activities of alanine aminotransferase (ALT) and aspartate aminotransferase (AST). The synergistic intervention resulted in the normalization of glucose-6-phosphatase and tyrosine aminotransferase activities, an elevation in hepatic glycogen stores, and a diminishment in triglyceride deposition, all while leaving hexokinase and citrate synthase activities unaltered. Furthermore, it reinstated the levels of ALT and AST, thereby suggesting a hepatoprotective effect. In summary, the combination of swimming exercise and (PhSe)₂ supplementation yielded an enhancement in hepatic glucose homeostasis among aged rats; however, further investigations are warranted to elucidate the mechanisms involved (52).

**Table 1:** Synergistic effects of diphenyl diselenide supplementation and exercise on neuroprotection, metabolic function, and inflammation in aging.

Animals	Intervention	Key findings	Conclusion	References
Male Wistar rats, 24 months old	1 ppm diphenyl diselenide (PhSe₂) in chow + swimming exercise (20 min/day for 4 weeks)	Combined treatment prevented age-related decreases in [^3^H]GABA uptake in cerebral cortex and striatum	Swimming exercise together with PhSe₂ supplementation modulates GABAergic neurotransmission and helps preserve cerebral cortical and striatal function in aged rats	(50)
Male Wistar rats, 27 months old	1 ppm diphenyl diselenide (PhSe₂) in chow + swimming exercise (1% body weight, 20 min/day for 4 weeks)	Combined treatment increased hippocampal neuroprotective proteins and reduced markers of apoptosis and neuroinflammation	Swimming exercise together with PhSe₂ supplementation promotes hippocampal neuroprotection and may counteract age-related cognitive decline	(51)
Male Wistar rats, old	1 ppm diphenyl diselenide (PhSe₂) in chow + swimming exercise (20 min/day, 5 days/week for 4 weeks)	Combined treatment normalized gluconeogenic enzyme activities, increased hepatic glycogen, reduced triglyceride accumulation, and restored ALT and AST levels	Swimming exercise with PhSe₂ supplementation improves hepatic glucose homeostasis and exerts hepatoprotective effects in aged rats	(52)
Male Wistar rats, 27 months old	1 ppm diphenyl diselenide (PhSe₂) in chow + swimming exercise (1% body weight, 20 min/day for 4 weeks)	Combined treatment decreased hypothalamic GFAP and Iba-1 levels, increased Bcl-2 and procaspase-3, reduced apoptotic markers (cleaved PARP/PARP, pJNK/JNK), and elevated mBDNF and NeuN levels	Swimming exercise with PhSe₂ supplementation promotes hypothalamic neuroprotection in aged rats by reducing apoptosis and glial cell activation	(53)
Male Wistar rats, 24 months old	1 ppm diphenyl diselenide (PhSe₂) in chow + swimming exercise (3% body weight, 20 min/day for 4 weeks)	Combined treatment improved short- and long-term memory, enhanced spatial learning, and increased hippocampal phosphorylated CREB levels, without affecting Akt	Swimming exercise with PhSe₂ supplementation enhances memory and cognitive function in aged rats by modulating cAMP/CREB signaling	(54)
Middle-age and old Wistar rats	1 ppm diphenyl diselenide (PhSe₂) in chow + swimming exercise (3% body weight, 20 min/day for 4 weeks)	In middle-age rats, combined treatment reduced pro-inflammatory cytokines (IL-1β, IL-6, TNF-α, IFN-*γ*) and increased IL-10; in old rats, PhSe₂ reversed the pro-inflammatory effects of swimming and increased IL-10	Swimming exercise and PhSe₂ supplementation modulate inflammatory responses in an age-dependent manner, exerting anti-inflammatory effects and mitigating age-related cytokine imbalance	(55)
Old male Wistar rats	1 ppm diphenyl diselenide (PhSe₂) in chow + swimming exercise (3% body weight, 20 min/day for 4 weeks)	Swimming alone or with PhSe₂ reduced hepatic ROS; only exercise improved GSH/GSSG ratio and prevented mitochondrial ROS elevation; combined treatment increased MnSOD but blunted some exercise-induced mitochondrial adaptations	Combining PhSe₂ with swimming does not produce a synergistic antioxidant effect in the liver and may attenuate certain exercise-induced mitochondrial benefits in aged rats	(56)

PhSe₂, diphenyl diselenide; ROS, reactive oxygen species; GSH, reduced glutathione; GSSG, oxidized glutathione; ALT, alanine aminotransferase; AST, aspartate aminotransferase; BDNF, brain-derived neurotrophic factor; CREB, cAMP-response element-binding protein; GFAP, glial fibrillary acidic protein; Iba-1, ionized calcium-binding adaptor molecule 1; PARP, poly(ADP-ribose) polymerase; JNK, c-Jun N-terminal kinase; NeuN, neuronal nuclei.

A research investigated the effects of a diet supplemented with PhSe₂, in conjunction with swimming exercise, on glial activation, apoptosis, and neuroprotective proteins within the hypothalamus of male rats aged 27 months. The process of aging was associated with an increase in astrocyte and microglial activation, a decrease in Bcl-2 and procaspase-3 levels, and an elevation in apoptotic markers, such as cleaved PARP and phosphorylated JNK (pJNK). Additionally, aged rats exhibited diminished levels of mature brain-derived neurotrophic factor (mBDNF), neuronal nuclear protein (NeuN), and the ratio of phosphorylated Akt to total Akt (pAkt/Akt). The combination of dietary and exercise interventions mitigated several of these alterations: it resulted in a reduction of glial fibrillary acidic protein (GFAP) and ionized calcium-binding adaptor molecule 1 (Iba-1), an increase in Bcl-2 and procaspase-3, and a decrease in apoptotic signaling pathways. Furthermore, it reinstated levels of mBDNF and NeuN, while the pAkt/Akt ratio remained unaffected. Collectively, the supplementation of PhSe₂ alongside swimming exercise facilitated neuroprotection in the aging hypothalamus by diminishing both apoptosis and glial cell activation (53). Another study examined whether combining a PhSe₂-supplemented diet with swimming exercise could improve memory in 24-month-old Wistar rats. Animals received standard chow or chow enriched with 1 ppm PhSe₂ for 4 weeks and performed daily swimming with a 3% body-weight workload. Cognitive performance was assessed using the object recognition and object location tests. The combined intervention enhanced short- and long-term memory as well as spatial learning. These improvements were accompanied by increased hippocampal phosphorylated CREB levels, indicating activation of memory-related signaling pathways. The findings indicated that PhSe₂ supplementation supports memory in aged rats by modulating cAMP and promoting CREB phosphorylation, while Akt levels remained unchanged (54). The purpose of study was to determine the influence of a diet supplemented with PhSe₂ and swimming exercise on serum cytokine concentrations in Wistar rats across various age groups. The process of aging was observed to elevate pro-inflammatory cytokines (IL-1β, IL-6, TNF-*α*, IFN-*γ*) while concurrently diminishing the levels of the anti-inflammatory cytokine IL-10. In the cohort of middle-aged rats, both interventions were found to significantly decrease pro-inflammatory cytokine levels and enhance IL-10 concentrations. Conversely, swimming as a standalone intervention exacerbated inflammatory responses in aged rats, whereas the dietary inclusion of PhSe₂ did not exhibit any significant independent effect. Nonetheless, when these two interventions were implemented in conjunction, the supplementation of PhSe₂ along with swimming exercise resulted in a reduction of pro-inflammatory cytokines and an increase in IL-10 levels across both age demographics. Collectively, the outcomes shown that both exercise and PhSe₂ exert modulatory effects on inflammatory responses in a manner that is dependent on age, with PhSe₂ demonstrating a capacity to mitigate exercise-induced inflammation in older subjects (55).

A research aimed to ascertain the effects of concomitantly administering PhSe₂ alongside swimming exercise on the mitigation of oxidative stress within hepatic tissue and mitochondrial structures of senescent rats. A cohort of aged male Wistar rats was systematically allocated into either sedentary or physically trained categories, with some receiving 1 ppm PhSe₂ over a duration of 4 weeks. The application of training, whether conducted independently or in conjunction with PhSe₂, resulted in a reduction of hepatic ROS, yet only the exercise regimen yielded a significant decrease in GSSG levels while concurrently enhancing the GSH/GSSG ratio. Notably, mitochondrial ROS levels were markedly elevated in the sedentary group and were exclusively ameliorated through the exercise intervention. Although the activity of manganese superoxide dismutase (MnSOD) was found to be elevated in the combined treatment group, PhSe₂ supplementation appeared to attenuate several exercise-induced mitochondrial adaptations, particularly those influencing membrane potential. In summary, the integration of PhSe₂ did not facilitate a synergistic antioxidant effect and indeed undermined certain beneficial outcomes of exercise concerning hepatic oxidative stress (56). Across the above empirical investigations, the synergistic application of PhSe₂ supplementation alongside swimming exercise consistently illustrates advantageous impacts on aging-associated physiological and neurological transformations in rat models. Collectively, these interventions augment neuroprotection through the upregulation of critical proteins such as BDNF, CREB, and Bcl-2, facilitating a reduction in apoptosis, diminishing glial activation, enhancing cognitive memory, and safeguarding neurotransmission via sustained GABA uptake. Furthermore, they positively influence metabolic and hepatic health by normalizing glucose-regulating enzymes, mitigating triglyceride accumulation, and reinstating ALT and AST levels to normative ranges. In addition, PhSe₂ and physical exercise modulate the expression of inflammatory cytokines in a manner contingent upon age, thereby mitigating exercise-induced inflammation in aged rats. Nevertheless, this synergy is not universally applicable, as PhSe₂ may occasionally attenuate exercise-induced mitochondrial adaptations within the hepatic tissue. Collectively, the results imply that the integration of PhSe₂ with physical exercise confers substantial protective benefits throughout the aging process, albeit with mechanisms that exhibit variability across different tissue types.

## 7 Clinical and preclinical evidence of amplifying effects of HMB combined with exercise in aging and age-related diseases

**Figure 2:**
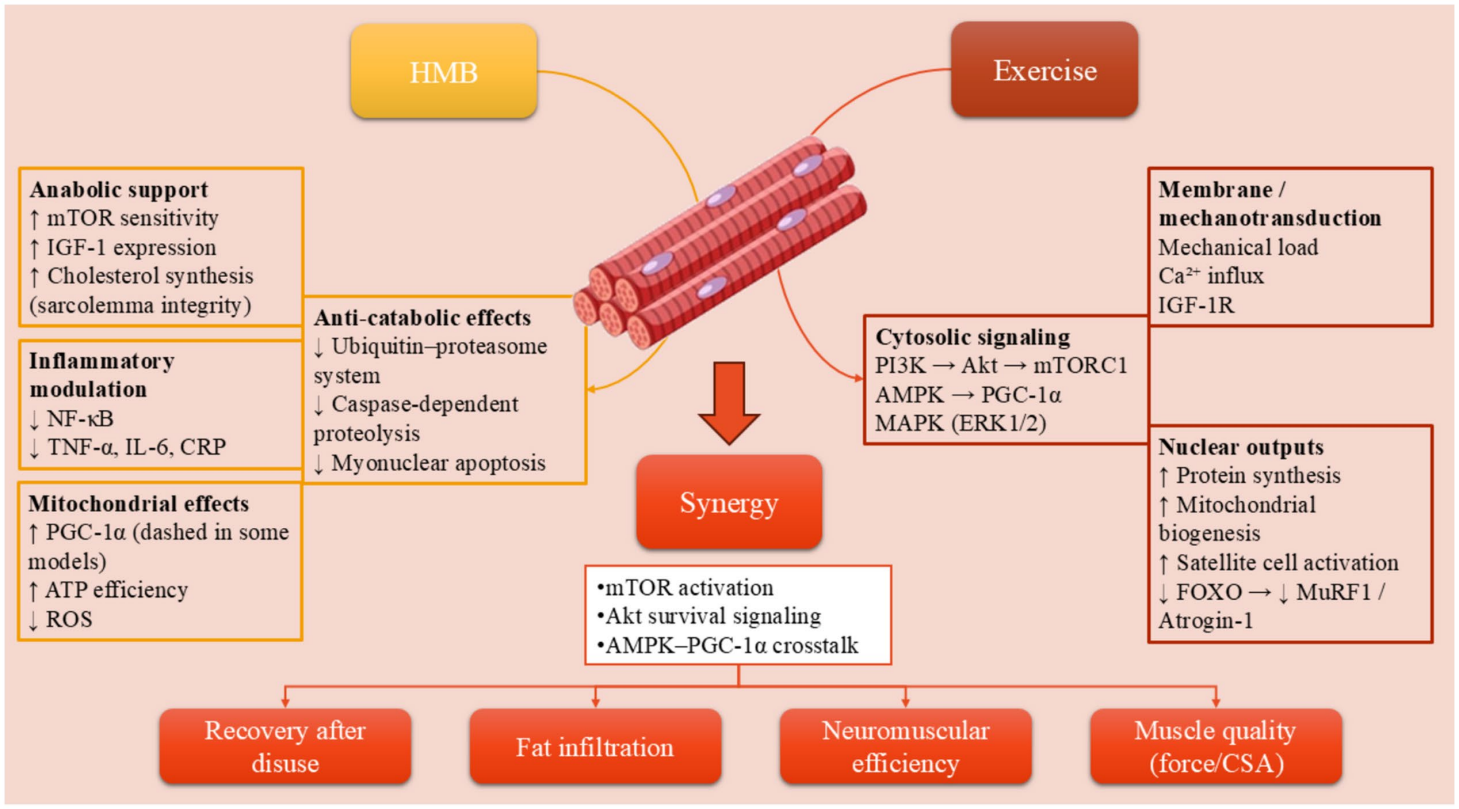
Amplifying effects of β-hydroxy-β-methylbutyrate (HMB) combined with exercise in aging. This figure summarizes how exercise and HMB interact to influence muscle and systemic aging pathways. Exercise is the primary anabolic stimulus, activating IGF-1/PI3K/Akt/mTOR, AMPK/PGC-1α, and MAPK signaling to enhance protein synthesis, mitochondrial biogenesis, and suppress atrophy pathways. HMB mainly amplifies and stabilizes these exercise-induced responses by increasing mTOR sensitivity, reducing proteolysis and apoptosis, and attenuating inflammatory signaling, with context-dependent effects on mitochondrial function. Convergent signaling improves muscle quality, strength, and resistance to age-related or disuse-induced decline. Evidence from animal models and older adults consistently supports benefits for physical function, while effects on muscle mass are variable and minimal in the absence of exercise.

Sarcopenia plays a pivotal role in the exacerbation of frailty and the deterioration of functional capabilities among the elderly population. An investigation assessed the impact of a 6-week regimen of creatine monohydrate (CRE) and HMB supplementation, in conjunction with an integral physical conditioning (IPC) protocol, on muscular strength and body composition in a sample of 30 physically active individuals aged 60 years and older. In a rigorously designed randomized, double-blind, placebo-controlled crossover trial, participants underwent two distinct 6-week interventions (CRE + HMB or placebo) alongside IPC training. The CRE + HMB supplementation resulted in a reduction of adipose tissue and a marginal enhancement of muscular parameters, whereas the placebo cohort exhibited contrasting trends. Notably, CRE + HMB markedly improved functional strength and endurance across various assessments, largely independent of alterations in muscle mass, thereby indicating a potential neuromuscular mechanism at play. This integrative strategy may facilitate the promotion of healthy aging (57) (Table 2 and Figure 2). A cluster-randomized trial examined the synergistic effects of physical exercise and HMB supplementation on cognitive and physical functioning, levels of disability, and muscle power among 72 institutionalized elderly individuals (mean age 83 ± 10 years; 63% female). Participants were allocated to one of four groups: exercise combined with placebo (EX), HMB supplementation alone, exercise supplemented with HMB (EX + HMB), or a control group (CT). Those in the exercise groups participated in a 12-week individualized multicomponent program (Vivifrail), while participants receiving HMB were administered a dosage of 3 g per day. Both the EX and EX + HMB cohorts demonstrated significant improvements in cognitive assessments, physical performance (as measured by the Short Physical Performance Battery, SPPB), and relative muscle power, in contrast to the control and HMB-only groups, which exhibited no significant changes. The findings suggested that HMB supplementation did not confer additional advantages, thereby underscoring the essential role of exercise as the primary factor in promoting health and functional capacity among very elderly adults (58).

**Table 2:** Effects of HMB supplementation with or without exercise on muscle mass, strength, and function in older adults.

Participants	Intervention	Duration	Main findings	Conclusion	References
30 older adults (20 men, 10 women; ≥60 y)	CRE + HMB or placebo + integral physical conditioning (strength, power, multicomponent circuits, HIIT, moderate-intensity training)	6 weeks per intervention period, 3-week washout	Fat mass ↓, Body fat % ↓, Muscle mass →, Functional strength ↑, Endurance ↑	CRE + HMB + IPC enhances functional strength and endurance, likely via neuromuscular mechanisms	(57)
72 institutionalized older adults (age 83 ± 10 y; 63% women)	12-week Vivifrail multicomponent exercise (5 days/week) ± 3 g/day HMB, plus control	12 weeks	Cognitive function ↑, Physical function ↑, Muscle power ↑, HMB alone →	Exercise improved cognitive and physical function and muscle power; HMB supplementation provided no additional benefit	(58)
Older adults with sarcopenia post-acute geriatric rehab (n not specified; women showed handgrip gains)	3 g/day Ca-HMB + 12-week resistance training (3 sessions/week) vs. placebo + same training	12 weeks (followed by 1-year follow-up)	SPPB-Balance ↑, Total SPPB ↑, SPPB-Chair Stand ↑, Handgrip strength ↑ (women), sustained at 1 year	HMB supplementation with exercise may enhance muscle strength and physical performance in older women with sarcopenia; further studies needed	(59)
Older adults ≥60 years with sarcopenia (HMB n = 18; placebo n = 16)	3 g/day HMB twice daily + resistance exercise 2x/week vs. placebo + same exercise	12 weeks	Handgrip strength ↑, Gait speed ↑, 5-time chair stand ↓, Muscle quality ↑, Inflammatory marker (TWEAK) ↓; Skeletal muscle mass =	HMB supplementation enhances the effects of resistance training on muscle strength, performance, and quality, and reduces inflammatory factors in older adults with sarcopenia	(60)
117 adults ≥60 y with low vitamin D	HMB + Vitamin D3 ± exercise; Control ± exercise	12 months	Lean body mass ↑ (nonexercisers) ↑, Knee extension peak torque ↑ (nonexercisers) ↑, Functional index ↑ (nonexercisers) ↑; No additive effect in exercising groups =	HMB + Vitamin D3 supplementation improves muscle strength and functional performance in older adults, particularly in those not performing exercise	(62)
104 adults ≥70 years, nursing home residents	4 groups: Ex-HMB (exercise + 3 g/day HMB), Ex-Plac (exercise + placebo), NoEx-HMB (HMB only), Controls (no exercise, no HMB); multicomponent exercise (VIVIFRAIL)	24 weeks	Functional capacity ↑ in exercise groups (Ex-HMB, Ex-Plac); Muscle strength ↑ in exercise groups; HMB alone showed minimal effect =; Cognitive function, frailty, body composition not significantly changed with HMB alone =	Multicomponent exercise improves functional outcomes in frail older adults; HMB supplementation alone adds negligible benefit when combined with exercise	(64)
16 healthy older men (HMB-FA: 67.8 ± 1.15 y; PLA: 68.5 ± 1.0 y)	HMB-FA (3 × 1 g/day) vs. placebo + unilateral RET (6 × 8 reps, 75% 1-RM, 3×/wk)	6 weeks	1-RM ↑ in both groups ↑; MVC ↑ in both groups ↑; VL thickness ↑ in both groups ↑; Thigh lean mass ↑ only in HMB-FA ↑; MPS early ↑ in trained leg with HMB-FA ↑, no difference at 4–6 wks =	HMB-FA did not significantly enhance muscle strength or mass over RET alone; early MPS and TLM gains suggest potential long-term benefit, but larger studies needed	(65)
48 men (66–78 y)	HMB only, RT only, HMB + RT, or placebo/no training	12 weeks	AFM ↓ in RT + HMB group vs. NT-PL, NT-HMB, RT-PL; no significant changes in other groups	HMB combined with resistance training effectively reduced abdominal fat mass in elderly men	(73)
≥65 y adults	Phase I: CaHMB only vs. placebo; Phase II: CaHMB + resistance exercise vs. placebo + resistance exercise	24 weeks	Phase I: LE60 ↑, MQ180 ↑, total LM ↑, leg LM ↑, LE180 ↑, FM ↓; Phase II: RE improved total LM ↑, LE60 ↑, LE180 ↑, HG ↑, GUG ↑; no additive effect of CaHMB with RE	CaHMB alone improved strength and muscle quality; resistance exercise improved all measures of body composition and functionality; no additive effect of CaHMB with exercise	(74)
156 women, 65–79 y, low muscle mass (SMI < 5.7 kg/m^2^)	2 × 2 factorial: Exercise-only, HMB-only (1,500 mg/day), both, or none	12 weeks + 12-week follow-up	Exercise ↑ usual & maximal gait speed, knee extensor & hip adductor strength, timed up-and-go, sit-to-stand; HMB ↑ usual gait speed slightly; no additive effect of HMB on other outcomes; most gains lost during follow-up	HMB provided minimal additional benefit; exercise was the primary effective intervention for improving physical function in older women with low muscle mass	(77)
50 women, mean age 76.7 y	Experimental: 2 g HMB + 5 g arginine + 1.5 g lysine/day; Placebo	12 weeks	↑ “get-up-and-go” performance (−2.3 s), ↑ limb circumference, ↑ leg & handgrip strength, ↑ whole-body protein synthesis (~20%), trend toward ↑ fat-free mass	Targeted nutritional supplementation with HMB, arginine, and lysine improved muscle functionality, strength, and protein synthesis in elderly women	(78)
16 healthy older men (Treatment *n* = 8, Placebo *n* = 8; Age ~68 y)	HMB supplementation (dose not specified) + unilateral resistance exercise training (6 × 8 reps at 75% 1-RM) vs. Placebo + same exercise	6 weeks	↑ 1-RM and MVC in both groups, ↑ thigh lean mass only in HMB group (NS between groups), ↑ VL thickness in exercised legs in both groups; no additive effect of HMB on muscle strength, function, or mass	HMB supplementation did not provide additional benefits over resistance training alone in healthy older men	(79)
59 older adults (Intervention *n* = 30, Placebo *n* = 28; Age 79.7 ± 4.8 y)	Dietary supplement containing HMB, carnosine, magnesium, butyrate, and lactoferrin vs. placebo; all participants maintained usual diet and activity	Short-term intervention	↑ Skeletal muscle index (SMI), ↑ muscle function (handgrip, chair, SPPB, walking speed), ↓ VAT, ↓ CRP, ↓ TNF-α, ↓ zonulin	The multi-component dietary supplement improved muscle mass, function, and markers of inflammation and gut health in older adults with sarcopenia	(80)
80 healthy older women (HMB *n* = 40, Control *n* = 40; 32 HMB and 33 control completed)	Oral supplement: 1.5 g calcium HMB daily vs. no supplement; all participated in twice-weekly mild fitness program	8 weeks	→ SPPB: no effect, → Handgrip strength: no effect, ↑ Peak torque isometric & isokinetic, ↑ 6MWT, ↑ handgrip endurance, ↑ muscle density (pQCT)	Calcium HMB improved muscle strength, endurance, and physical performance, but did not affect SPPB or overall body composition.	(81)

HMB, β-hydroxy-β-methylbutyrate; CRE, creatine monohydrate; IPC, integral physical conditioning; SPPB, Short Physical Performance Battery; PT, peak torque; 6MWT, 6-min walk test; DXA, dual-energy X-ray absorptiometry; pQCT, peripheral quantitative computerized tomography; RET, resistance exercise training; TLM, thigh lean mass; MVC, maximal voluntary contraction; RW, running wheel; AFM, abdominal fat mass; FM, fat mass; LM, lean mass; MQ, muscle quality; SC, satellite cells; MN, myonuclei; MPS, muscle protein synthesis; FA, fractional anisotropy; TNF-α, tumor necrosis factor-alpha; CRP, C-reactive protein; VAT, visceral adipose tissue; FSMP, food for special medical purposes; β-Ala, beta-alanine.

**↑**, Increase/Improvement; **↓**, Decrease/Reduction; ↔, No effect/No change/Neutral; =, Equal/Same/No difference.

A randomized, double-blind, placebo-controlled investigation assessed the ramifications of incorporating 3 g/day of Ca-HMB supplementation into a 12-week resistance training regimen among older adults exhibiting sarcopenia subsequent to acute rehabilitation. Participants were allocated to either the HMB plus exercise group or the placebo plus exercise group. The principal outcomes encompassed handgrip strength, the Short Physical Performance Battery (SPPB), and 4-meter gait velocity, evaluated at baseline, following the intervention, and at a 1-year follow-up. The intervention cohort exhibited statistically significant enhancements in the total SPPB score, balance, and chair-stand performance, with handgrip strength improvements in females preserved over the 1-year period. The observations indicated that the combination of Ca-HMB and resistance training may augment muscle strength and physical functionality in older women; however, additional research is requisite to substantiate its efficacy (59). The aim of study was to investigate the ramifications of a 12-week HMB supplementation in conjunction with resistance training among 34 elderly individuals (≥60 years) exhibiting sarcopenia. Participants were allocated to either the HMB or placebo cohorts and engaged in training sessions biweekly. The principal and secondary endpoints encompassed metrics such as handgrip strength, gait velocity, five-time chair stand performance, body composition, muscle quality, and inflammatory biomarkers. In comparison to the placebo group, HMB administration substantially enhanced handgrip strength, gait velocity, chair-stand performance, and muscle quality, while concurrently diminishing tumor necrosis factor-like weak inducer of apoptosis; however, skeletal muscle mass and other body composition parameters remained constant. HMB augments the advantages of resistance exercise concerning strength, performance, and inflammation, thereby endorsing its prospective utility as an intervention for sarcopenia (60).

The process of aging is associated with a decrement in both muscle mass and strength; however, consistent engagement in physical exercise has the potential to sustain muscle health. HMB and black ginger (BG) have been shown to enhance muscle protein metabolism and energy production, possibly resulting in synergistic effects. The purpose of another study was to examine 28-week-old SAMP8 murine models, which were categorized into six distinct groups, including exercise alone, exercise in conjunction with HMB, BG, or a combination of both (Ex+Comb), over a duration of 16 weeks. Although the implementation of exercise with HMB or BG alone did not yield significant improvements, the Ex+Comb cohort demonstrated an increase in muscle mass and strength, augmented expression of genes associated with mitochondrial function and biogenesis, as well as modulation of autophagy-related proteins (Atg3, 7, 16 L1, Beclin1). The integration of long-term habitual exercise with HMB and BG may enhance muscle health through the activation of mitochondrial and autophagy pathways (61) (Table 3). A 12-month, randomized, double-blind investigation assessed the impact of calcium HMB and vitamin D3 (D) supplementation on muscular strength and functional capacity in 117 senior individuals (aged over 60 years) exhibiting deficient levels of 25-hydroxy-vitamin D. Subjects were systematically allocated to either a control group or an HMB + D group, with variations in exercise participation. Among those not engaging in exercise, HMB + D supplementation resulted in significant enhancements in lean body mass, knee extension peak torque, and a composite functional index at the 3, 6, and 12-month intervals when compared to control subjects. No supplementary advantages were recorded among participants who engaged in exercise. These outcomes demonstrated that HMB + D supplementation possesses the potential to improve muscular strength and physical functionality in elderly populations, even in the absence of formalized exercise regimens (62).

**Table 3:** Effects of HMB supplementation with or without exercise on muscle mass, strength, and function in animal models.

Participants	Intervention	Duration	Main findings	Conclusion	References
12 aged female Sprague–Dawley rats (19 mo)	HMB 0.46 g/kg/d vs. no HMB; both groups performed ladder-climbing RT every 3 days	10 weeks	→ Neuromuscular functions: no effect, → Myofiber dimension: no effect, → Satellite cells & myonuclei: no effect, ↑ Fat mass reduction in HMB group	HMB did not provide additive benefits over intense RT, but may help reduce fat mass; RT alone was sufficient to improve muscle composition and function.	(82)
19-mo female Sprague–Dawley rats	HMB (0.48 g/kg/d) + RT vs. RT only vs. control	10 weeks	Whole body strength ↑, grip strength ↑, lean mass ↑, fat mass ↓, myofiber CSA ↑, satellite cells ↑, myonuclei ↑; IGF-I mRNA ↑ only in soleus of HMB group; MGF and myogenin ↑ in both RT groups	HMB did not provide additional benefits beyond intense RT; RT alone effectively enhanced muscle strength, mass, and regenerative markers	(75)
34-mo Fisher 344 × Brown Norway rats	Ca-HMB (340 mg/kg) vs. water vehicle	14 days hindlimb suspension + 14 days reloading	Maximal isometric force ↑ during recovery; plantaris and soleus fiber CSA ↑; TUNEL-positive nuclei ↓; Bax protein ↓; cleaved caspase-3 & -9 ↓; Bcl-2 unchanged	HMB attenuated disuse-induced muscle loss and myonuclear apoptosis, promoting recovery after unloading, but did not fully prevent atrophy	(76)
20-month-old Sprague–Dawley rats (n = 10/group)	HMB + β-Alanine supplementation vs. control + voluntary running wheels	4 weeks (after 4-week baseline)	Muscle CSA trend ↑; Muscle force/fatigue resistance =; Muscle quality ↓; PGC1-α ↓; LC3-II/I ratio ↓; RW activity in experimental group ↓	Short-term HMB + β-Ala with modest exercise provided minimal improvement in muscle function; some molecular markers of mitochondrial biogenesis and autophagy were reduced	(72)
Aged male Fisher 344 × Brown Norway rats (34–36 mo.)	Daily gavage with 170 mg Ca-HMB or water; hindlimb suspension (HS) for 14 days followed by 14 days reloading	28 days	Body weight ↓ (~18% after HS, ~22% after R) ↑; EDL muscle mass and fiber CSA =; Twitch contraction, force, fatigue resistance =; Apoptotic signaling (Bax, BCL-2, TUNEL) ↑; Ca-HMB had no effect =	Ca-HMB did not improve muscle mass, function, or apoptotic signaling in aged rats when muscle atrophy was not induced	(63)
Senescence-accelerated mice (SAMP8), 28–44 weeks old	Exercise alone, HMB alone, BG alone, Exercise + HMB + BG	16 weeks	Muscle mass ↑, Muscle strength ↑, Mitochondrial function genes ↑, Autophagy-related proteins altered ↑ (Ex+HMB + BG); Exercise or HMB/BG alone =	Habitual exercise combined with HMB and black ginger synergistically enhances muscle mass, strength, mitochondrial function, and autophagy in aging mice	(61)

mo, months; HMB, β-hydroxy-β-methylbutyrate; RT, resistance training; CSA, cross-sectional area; IGF-I, insulin-like growth factor I; MGF, mechano growth factor; Ca-HMB, calcium HMB; TUNEL, terminal deoxynucleotidyl transferase dUTP nick end labeling; Bax, Bcl-2-associated X protein; Bcl-2, B-cell lymphoma 2; β-Ala, beta-alanine; PGC1-α, peroxisome proliferator-activated receptor gamma coactivator 1-alpha; LC3-II/I, microtubule-associated protein 1A/1B-light chain 3 II/I ratio; EDL, extensor digitorum longus; HS, hindlimb suspension; BG, black ginger; Ex, exercise.

**↑**, Increase/Improvement, **↓**, Decrease Reduction; ↔, No effect/No change/Neutral; =, Equal/Same/No difference.

A study determined the potential impact of calcium (Ca-HMB) on muscle functionality and apoptotic signaling within the extensor digitorum longus (EDL) of senescent rats subjected to hindlimb suspension (HS) and subsequent reloading (R) without the occurrence of muscle atrophy. Aged rats were administered Ca-HMB or a control solution prior to, throughout, and subsequent to a 14-day period of HS, followed by a 14-day period of R. Notwithstanding considerable reductions in body weight, parameters such as EDL mass, fiber cross-sectional area, force generation, and resistance to fatigue exhibited no significant alterations. Apoptotic signaling was found to be elevated during both HS and R; however, the administration of Ca-HMB did not alter these signaling pathways. These results exhibited that Ca-HMB does not enhance muscle performance or mitigate apoptotic signaling in the absence of atrophic conditions (63). The HEAL (HMB + Exercise = Adults Living longer) study examines the synergistic effects of multicomponent physical activity and HMB supplementation on the health outcomes of frail elderly individuals. In a 24-week investigation, a cohort of 104 adults aged 70 years and above residing in nursing facilities is systematically allocated into four distinct groups: those engaging in exercise supplemented with HMB, those participating in exercise with a placebo, those receiving HMB supplementation exclusively, and a control group. Participants in the exercise groups undertake the tailored VIVIFRAIL program, while individuals in the HMB groups are administered a dosage of 3 g/day. The principal outcome measure is functional capacity, whereas secondary outcome measures encompass muscle strength, levels of frailty, body composition, cardiometabolic risk factors, cognitive performance, and depressive symptoms. The outcomes were anticipated to inform the development of cost-effective interventions aimed at improving health and overall quality of life among older adults (64). Another research investigation examined the efficacy of chronic HMB-FA supplementation in augmenting muscle protein synthesis (MPS), muscle mass, and functional capacity in a cohort of 16 healthy older males participating in a 6-week regimen of unilateral resistance exercise training (RET). Both the HMB-FA and placebo cohorts demonstrated enhancements in strength, vastus lateralis thickness, and maximal voluntary contraction, with no discernible differences observed between the groups. A significant increase in thigh lean mass was exclusively noted in the HMB-FA group, which was corroborated by early elevations in MPS; however, overall MPS and myogenic gene expression did not exhibit any differences across the groups. The data exhibited that HMB-FA may provide a modest augmentation of lean mass in older males, while RET alone predominantly facilitates the primary adaptations in strength and muscle; further longitudinal studies are warranted to validate any functional advantages (65).

Research has demonstrated that *β*-alanine (β-Ala) supplementation can improve submaximal exercise capacity in both generally healthy but relatively inactive older adults and master-level athletes, likely through its role in increasing muscle carnosine content and enhancing intramuscular buffering capacity (66, 67). Animal studies further support these findings, showing that *β*-Ala supplementation enhances fatigue resistance and physical performance in rodent models (68–70). In parallel, emerging evidence highlights the role of amino acids such as L-leucine, a precursor of HMB, in modulating neuroprotective pathways in AD models, including improvements in metabolic parameters (71). The aim of study was to determine the impacts of co-supplementation with HMB and *β*-Ala on muscular functionality in aged (20 months) Sprague–Dawley rats granted access to voluntary running wheels. The subjects were allocated to either a control group or a diet comprised of HMB + β-Ala for a duration of 4 weeks. The administration of HMB + β-Ala did not yield significant improvements in muscle strength or endurance against fatigue, and although a tendency towards an augmentation in muscle cross-sectional area was noted, there was a concomitant decline in muscle quality (force/CSA). Supplementation resulted in a reduction of indicators associated with mitochondrial biogenesis (PGC1-*α*) and autophagy (LC3-II/I ratio) and influenced myostatin signaling pathways. Interestingly, an increase in running activity was observed within the control group, while the experimental groups did not exhibit similar trends. In summary, the short-term application of HMB + *β*-Ala conferred negligible advancements in muscular functionality among healthy aging rats (72). A research explored the impact of a 12-week regimen of HMB supplementation in conjunction with resistance training (RT) on abdominal fat mass (AFM) among 48 male subjects aged 66 to 78 years. Participants were systematically allocated into one of four experimental groups: no-training placebo (NT-PL), HMB only (NT-HMB), RT with placebo (RT-PL), or RT with HMB (RT-HMB). The assessment of AFM was conducted using dual-energy X-ray absorptiometry (DXA) across the L1–L4 vertebral region. The analysis of covariance (ANCOVA) indicated significant discrepancies in the adjusted posttest AFM, with pairwise comparisons revealing that the RT-HMB cohort exhibited a significantly reduced AFM compared to NT-PL, NT-HMB, and RT-PL. These results demonstrated that the synergistic application of HMB alongside RT is effective in diminishing abdominal fat in elderly males (73).

A study examined the ramifications of a 24-week regimen of calcium CaHMB supplementation, with or without concomitant resistance exercise (RE), in a cohort of individuals aged 65 years or older. Phase I of the study juxtaposed the effects of CaHMB against a placebo in the absence of exercise, whereas Phase II scrutinized the impact of RE alongside either CaHMB or placebo supplementation. In Phase I, the administration of CaHMB was found to significantly enhance leg extension strength, muscle quality, and lean body mass in comparison to the placebo group. Phase II revealed that RE significantly augmented total lean mass, leg strength, handgrip strength, and functional performance, irrespective of the supplementation regimen. Notably, CaHMB administered in isolation resulted in a reduction of fat mass and an enhancement of muscle quality. Cumulatively, the data shown that CaHMB contributes positively to strength and muscle quality among sedentary older adults, whereas RE serves to effectively ameliorate body composition and functional performance outcomes (74).

The purpose of study was to determine whether HMB augments muscle adaptations induced by resistance training (RT) in aged female rats (19 months of age). The subjects were allocated into HMB, non-HMB, or control cohorts, with resistance training conducted every third day over a duration of 10 weeks. Both resistance training cohorts exhibited significant enhancements in whole-body strength, grip strength, lean body mass, as well as a reduction in fat mass. The cross-sectional area of myofibers within the gastrocnemius and soleus muscles increased by 8–22%, accompanied by an elevation in the numbers of satellite cells and myonuclei within the soleus muscle. HMB specifically induced an elevation in IGF-I mRNA levels in the soleus, while both resistance training groups demonstrated increases in MGF and myogenin expression. In summary, HMB did not provide any additional enhancement to the myogenic responses or muscle hypertrophy induced by resistance training in aged female rats (75). Another work assessed the impact of HMB on muscle atrophy and recovery subsequent to disuse in 34-month-old Fisher 344 × Brown Norway rats. The subjects were administered either Ca-HMB or a control solution during a period of 14 days of hindlimb suspension (HS) and/or 14 days of subsequent reloading (R). HMB effectively mitigated reductions in maximal isometric force and enhanced the cross-sectional area of plantaris and soleus muscle fibers following reloading. Furthermore, HMB significantly diminished the presence of TUNEL-positive nuclei and reduced levels of pro-apoptotic proteins (Bax, cleaved caspase-3, and caspase-9), while Bcl-2 expression remained stable. Although HMB did not completely avert the atrophy associated with unloading, it lessened the loss of muscle fibers in both fast and slow muscle types, presumably by inhibiting myonuclear apoptosis via mitochondrial caspase pathways, thereby reinforcing its protective function against muscle degeneration (76).

A 12-week intervention investigated the efficacy of HMB supplementation in augmenting the effects of exercise on muscle mass, strength, and physical performance among 156 older female participants aged 65 to 79 years with diminished muscle mass. The participants were allocated to one of four groups: exercise-only, HMB-only, combined intervention, or control, followed by a 12-week observational phase. Engagement in exercise yielded significant improvements in gait velocity, strength of knee extensors and hip adductors, as well as performance metrics such as timed up-and-go and sit-to-stand tasks. HMB supplementation resulted in a modest enhancement in usual gait velocity; however, it did not amplify the exercise-related benefits observed in other performance outcomes. The majority of the improvements were found to regress during the follow-up period, suggesting that exercise remains the principal and most effective strategy for enhancing functional capabilities in older women with inadequate muscle mass (77). A study evaluated whether a nutritional mixture of HMB, arginine, and lysine improves muscle health in elderly women (mean age 76.7 years). Participants were randomized to receive either placebo or the experimental mixture daily for 12 weeks. The experimental group showed a 17% improvement in the “get-up-and-go” test, along with increases in limb circumference, leg strength, handgrip strength, and positive trends in fat-free mass. Whole-body protein synthesis increased by ~20% compared with placebo. These results shown that targeted supplementation with HMB, arginine, and lysine enhances muscle function, strength, fat-free mass, and protein synthesis in elderly women, supporting its potential for improving muscle health (78).

A 6-week randomized, double-blind, placebo-controlled investigation examined the effects of HMB supplementation in conjunction with unilateral resistance exercise training (RET) among sixteen healthy older males, with a mean age of approximately 68 years. Both the HMB and placebo cohorts demonstrated significant enhancements in one-repetition maximum (1-RM) strength, maximal voluntary contraction, vastus lateralis thickness, and thigh lean mass; however, statistical significance regarding thigh lean mass was exclusively observed in the HMB group. No noteworthy disparities were detected between the groups for any measured parameter revealed alterations solely in cMyc gene expression. These results demonstrated that, within a community-dwelling population of older males, HMB supplementation fails to confer any supplementary advantages in muscle strength, mass, or functional capacity beyond that provided by RET alone (79). The aim of research was to assess the impact of a dietary supplement (DS) comprising HMB, carnosine, magnesium, butyrate, and lactoferrin on muscle mass, functionality, inflammation, and gastrointestinal health among 59 elderly individuals (mean age 79.7 years). Participants were allocated to either the DS group (*n* = 30) or the placebo group (*n* = 28) through a randomization process. The group receiving the supplement demonstrated notable enhancements in skeletal muscle index, a decrease in visceral adipose tissue, and improved performance in handgrip, chair stand, SPPB, and walking speed assessments. Inflammatory biomarkers (CRP, TNF-α) and the gut permeability marker (zonulin) exhibited significant reductions. These outcomes indicated that specific supplementation may facilitate increases in muscle mass and functionality while concurrently modulating inflammatory responses and the gut-muscle axis in sarcopenic older adults (80).

A 8-week intervention scrutinized the impacts of 1.5 g/day calcium HMB supplementation on physical performance and muscular strength in a cohort of 80 healthy older women engaged in mild fitness regimens. The primary endpoint, as measured by the Short Physical Performance Battery (SPPB) score, did not demonstrate statistically significant divergences between the intervention and control groups. Nonetheless, HMB supplementation resulted in a notable enhancement in peak torque isometric and isokinetic strength of the lower extremities, six-minute walking distance, handgrip endurance, and muscle density as evaluated through peripheral quantitative computed tomography (pQCT). Body composition assessments conducted via DXA exhibited no significant alterations. No serious adverse events were recorded throughout the trial duration. These findings demonstrated that HMB supplementation may facilitate improvements in muscular strength and specific physical performance metrics in healthy elderly female subjects, notwithstanding its limited influence on overall functional assessment scores (81). Another research aimed to elucidate whether HMB supplementation augments the outcomes of a 10-week resistance training (RT) regimen on muscle and body composition in 19-month-old female Sprague–Dawley rats. The subjects were systematically allocated to either HMB or non-HMB cohorts and underwent a ladder climbing training protocol every 3 days. Both cohorts exhibited substantial enhancements in neuromuscular function, myofiber cross-sectional area, satellite cell proliferation, myonuclear content, and regenerative potential, with no statistically significant disparities observed between the HMB and non-HMB groups. HMB supplementation did not confer additional advantages beyond those attained through RT, albeit it did facilitate a reduction in adipose tissue mass. These data indicated that rigorous RT alone is efficacious in promoting muscle hypertrophy, functional capacity, and regenerative processes in aged female rats, with only marginal contributions to fat loss attributable to HMB supplementation (82). Overall, HMB supplementation has been extensively investigated for its efficacy in preserving muscle mass, strength, and functional capacity in elderly populations as well as in animal models. Across various clinical trials, HMB consistently demonstrated enhancements in muscle strength, endurance, and functional performance metrics, such as leg and arm strength, chair stand transitions, gait velocity, and timed up-and-go assessments, albeit the increases in muscle mass were frequently modest. When integrated with resistance or multicomponent exercise regimens, HMB significantly augmented functional outcomes, yielding only minor additive effects on muscle hypertrophy. Furthermore, it contributed to reductions in adipose tissue, mitigated inflammatory responses, and modulated pathways associated with apoptosis and autophagy within muscular tissues. In animal studies, HMB was shown to enhance myofiber regeneration, improve mitochondrial functionality, and elevate muscle quality, especially when combined with exercise or other bioactive agents. Certain research indicated that the majority of benefits were attributable to exercise alone, while HMB conferred supplementary enhancements. In summary, HMB is deemed safe and exhibits maximal efficacy when utilized in conjunction with structured exercise, thereby presenting a promising nutritional strategy for the attenuation of sarcopenia, the enhancement of muscle functionality, and the support of healthy aging.

## 8 Future perspectives and therapeutic implications

The integrative application of PhSe₂, HMB, and physical exercise constitutes a promising multifaceted approach for alleviating age-associated declines in neurocognitive and musculoskeletal health. Subsequent investigations ought to concentrate on refining the dosage, timing, and synergistic combinations of PhSe₂ and HMB to optimize their combined efficacy while ensuring the safety of elderly populations. Preliminary investigations indicate that the concurrent administration of these compounds may enhance mitochondrial functionality, diminish oxidative stress, and modulate inflammatory pathways; however, the ideal concentrations and duration of interventions have yet to be determined. The prospects of multifaceted interventions in the context of aging are particularly intriguing. Aging represents a complex, multifactorial phenomenon characterized by oxidative stress, inflammation, mitochondrial dysfunction, along with the deterioration of muscle mass and functionality. Interventions that concurrently target these biological pathways, such as the supplementation of PhSe₂ and HMB in conjunction with exercise, have the potential to yield comprehensive benefits, including enhanced neuroprotection, improved cognitive function, and the preservation of physical performance. Guidelines for clinical investigations and individualized interventions ought to prioritize meticulously structured, longitudinal research involving heterogeneous elderly cohorts. Stratification predicated on initial cognitive functioning, sarcopenia, and metabolic health status may permit the tailoring of supplementation and exercise protocols. The incorporation of biomarkers indicative of oxidative stress, inflammation, and mitochondrial functionality will further enhance treatment methodologies and promote the translatability of preclinical insights into clinical practice. Collectively, this integrative strategy signifies a promising pathway for fostering healthy aging and alleviating the decline in functional capabilities associated with aging.

A key challenge in designing combined exercise and nutraceutical interventions for older adults is the high burden of comorbidities such as prediabetes, type 2 diabetes, and hypertension. These conditions are not simply concurrent diagnoses; they are biologically linked to core aging processes, including mitochondrial impairment, chronic low-grade inflammation, elevated oxidative stress, and reduced anabolic signaling. For example, insulin resistance and elevated blood glucose can impair mitochondrial function and increase reactive oxygen species, which may influence the redox-modulating actions of PhSe₂. Likewise, the chronic inflammatory state and endothelial dysfunction common in hypertension could dampen the vascular and neuroprotective benefits that typically accompany exercise. In the case of HMB, the anabolic resistance frequently present in metabolic disease may alter its ability to promote muscle protein synthesis and limit muscle breakdown. Despite these important interactions, most of the studies reviewed here did not stratify participants by comorbidity status, reducing the applicability of their findings to real-world aging populations. Furthermore, medications routinely used to manage these conditions such as glucose-lowering therapies or antihypertensive drugs, may themselves influence metabolic and inflammatory pathways, potentially modifying responses to both supplements and exercise. Future studies should therefore include detailed characterization of participants’ metabolic and cardiovascular profiles, medication use, and disease severity to better understand how comorbidities shape treatment responsiveness. Such efforts will support the development of targeted, multimodal strategies that optimize safety and effectiveness in diverse, clinically complex older adults.

Body mass index (BMI), along with body composition in particular, is another important factor influencing the effectiveness of exercise and nutraceutical interventions in older adults. While BMI is commonly used as a clinical indicator, it does not differentiate between fat mass and lean mass, which have distinct implications for metabolic function and physical performance. With aging, body composition often shifts in unfavorable ways, including the development of sarcopenia, increased visceral fat accumulation, or the combination of both conditions known as sarcopenic obesity. These states are linked to pro-inflammatory profiles, decreased insulin sensitivity, mitochondrial dysfunction, and reduced anabolic responsiveness to exercise. In individuals with higher adiposity, persistent low-grade inflammation and oxidative stress may reduce the potential benefits of compounds such as PhSe₂ and HMB, possibly necessitating modified dosing or combined intervention strategies. In contrast, older adults with sarcopenia and reduced muscle mass may experience greater benefits from HMB supplementation, particularly when it is paired with resistance exercise to support muscle protein turnover and maintain functional capacity. Additionally, body composition may affect the pharmacokinetics and tissue distribution of lipophilic molecules like PhSe₂, thereby influencing their biological effects. Despite the relevance of these factors, many studies assessing such interventions rely primarily on BMI rather than detailed body composition analysis. Future research should therefore incorporate more precise assessment methods, such as DXA or bioelectrical impedance analysis, to better characterize participant profiles. Including these measures in study design would facilitate more personalized intervention approaches and enhance the applicability of findings for improving metabolic and musculoskeletal health in heterogeneous aging populations.

## 9 Conclusion

**Figure 3:**
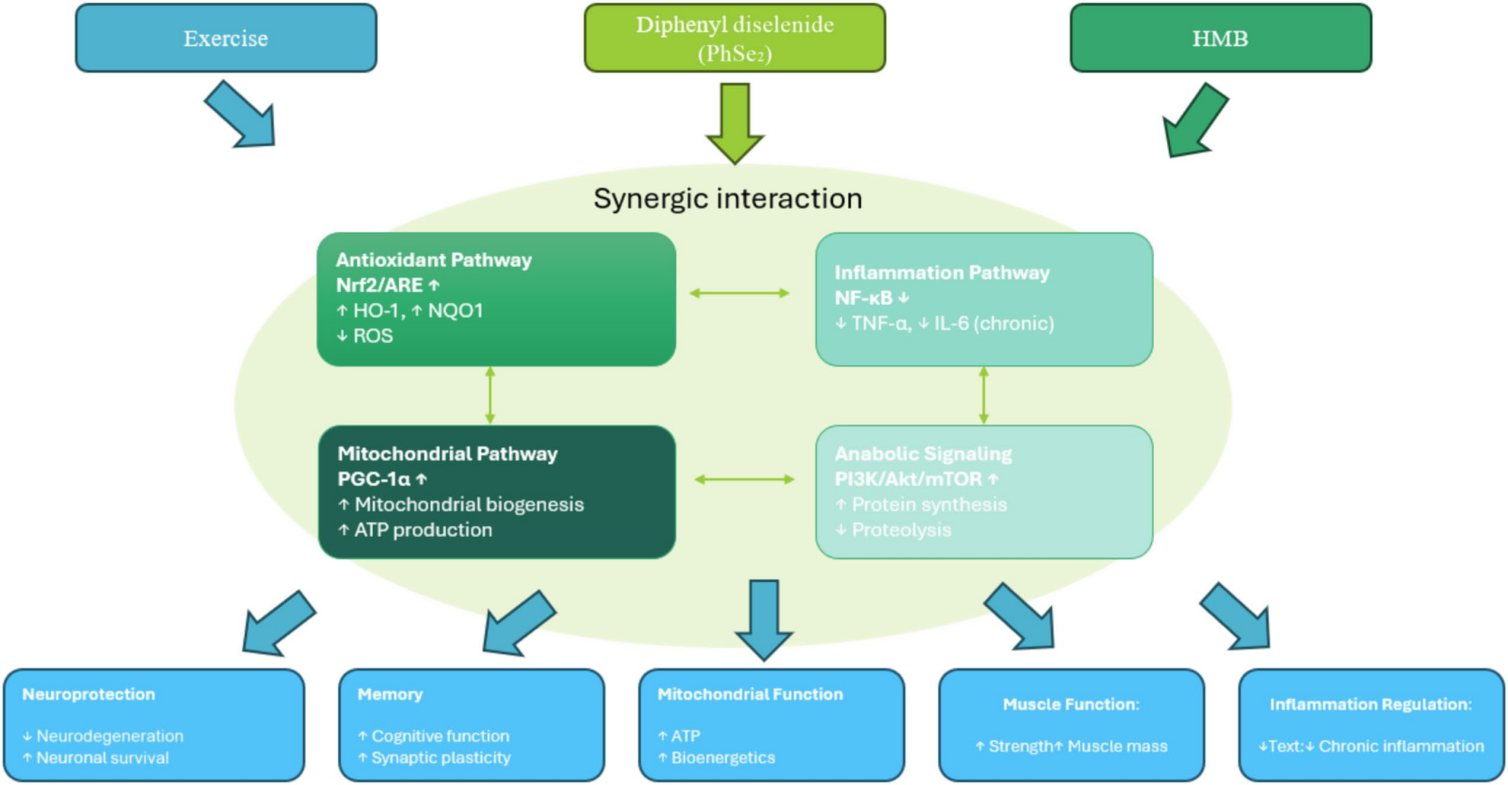
Synergistic effects of exercise, diphenyl diselenide (PhSe_2_), and β-hydroxy-β-methylbutyrate (HMB) on cellular and functional outcomes. This schematic illustrates the synergistic effects of exercise, PhSe_2_, and HMB on critical molecular pathways and physiological functions. Together, they enhance antioxidant defenses through the Nrf2/ARE pathway, increasing HO-1 and NQO1 levels while reducing ROS, and modulate inflammation via the NF-κB pathway, decreasing chronic TNF-α and IL-6 expression. They promote mitochondrial health through PGC-1α activation, leading to increased mitochondrial biogenesis and ATP production, and stimulate anabolic signaling via the PI3K/Akt/mTOR pathway, enhancing protein synthesis and reducing proteolysis. These molecular adaptations collectively improve neuroprotection by reducing neurodegeneration and supporting neuronal survival, enhance memory through increased cognitive function and synaptic plasticity, optimize mitochondrial function and bioenergetics, support muscle function by increasing strength and mass, and regulate chronic inflammation.

The integration of PhSe₂, HMB, and structured physical activity exhibits considerable synergistic potential in facilitating the process of healthy aging (Figure 3). Empirical evidence derived from both preclinical and clinical investigations suggests that the concurrent application of these interventions can significantly enhance neuroprotection, cognitive function, and mitochondrial physiology, whilst also augmenting muscle mass, strength, and overall physical performance. Furthermore, this combinatorial strategy is instrumental in modulating inflammatory pathways and mitigating oxidative stress, thereby addressing various hallmarks associated with aging and age-related pathologies. Important insights for the management of aging and age-related diseases encompass: (i) exercise continues to be a fundamental intervention, with the addition of PhSe₂ and HMB potentially magnifying its advantageous effects; (ii) concurrently targeting multiple physiological systems (neurological, muscular, and metabolic) may yield superior outcomes relative to isolated interventions; and (iii) tailored strategies, guided by biomarkers indicative of oxidative stress, inflammation, and mitochondrial function, have the potential to optimize individual responses and enhance therapeutic efficacy. In summary, the incorporation of PhSe₂ alongside HMB supplementation within an exercise regimen constitutes a potentially efficacious, multi-faceted approach aimed at alleviating cognitive and musculoskeletal deterioration associated with aging, thereby improving overall quality of life and diminishing susceptibility to age-related pathologies. Subsequent investigations, particularly those involving rigorously designed clinical trials, are essential to determine optimal intervention protocols and to effectively translate these findings into applicable strategies for the elderly population.

## References

[1] Franceschi C Garagnani P Parini P Giuliani C Santoro A. Inflammaging: a new immune-metabolic viewpoint for age-related diseases. Nat Rev Endocrinol. (2018) 14:576–90. doi: 10.1038/s41574-018-0059-4, 30046148

[2] Cicali KA Torres AK Tapia-Rojas C. Synaptic mitochondria in aging and neurodegenerative diseases: functional decline and vulnerability. Neural Regen Res. (2026) 21:2145–52. doi: 10.4103/NRR.NRR-D-24-01571, 40536922 PMC13211840

[3] Cuestas Torres DM Cardenas FP. Synaptic plasticity in Alzheimer’s disease and healthy aging. Rev Neurosci. (2020) 31:245–68. doi: 10.1515/revneuro-2019-0058, 32250284

[4] Bagetta V Ghiglieri V Sgobio C Calabresi P Picconi B. Synaptic dysfunction in Parkinson's disease. Biochem Soc Trans. (2010) 38:493–7. doi: 10.1042/BST0380493, 20298209

[5] Liguori I Russo G Curcio F Bulli G Aran L Della-Morte D . Oxidative stress, aging, and diseases. Clin Interv Aging. (2018) 13:757–72. doi: 10.2147/CIA.S158513, 29731617 PMC5927356

[6] Haas RH. Mitochondrial dysfunction in aging and diseases of aging. Biology. (2019) 8:48. doi: 10.3390/biology8020048, 31213034 PMC6627182

[7] Pedersen BK Febbraio MA. Muscles, exercise and obesity: skeletal muscle as a secretory organ. Nat Rev Endocrinol. (2012) 8:457–65. doi: 10.1038/nrendo.2012.49, 22473333

[8] Hamilton GF Rhodes JS. Exercise regulation of cognitive function and neuroplasticity in the healthy and diseased brain. Prog Mol Biol Transl Sci. (2015) 135:381–406. doi: 10.1016/bs.pmbts.2015.07.004, 26477923

[9] Jimoh YA Lawal AO Kade IJ Olatunde DM Oluwayomi O. Diphenyl diselenide modulates antioxidant status, inflammatory and redox-sensitive genes in diesel exhaust particle-induced neurotoxicity. Chem Biol Interact. (2022) 367:110196. doi: 10.1016/j.cbi.2022.110196, 36174737

[10] Duan Y Li F Li Y Tang Y Kong X Feng Z . The role of leucine and its metabolites in protein and energy metabolism. Amino Acids. (2016) 48:41–51. doi: 10.1007/s00726-015-2067-1, 26255285

[11] Wilkinson DJ Hossain T Hill DS Phillips BE Crossland H Williams J . Effects of leucine and its metabolite β-hydroxy-β-methylbutyrate on human skeletal muscle protein metabolism. J Physiol. (2013) 591:2911–23. doi: 10.1113/jphysiol.2013.253203, 23551944 PMC3690694

[12] Fitschen PJ Wilson GJ Wilson JM Wilund KR. Efficacy of β-hydroxy-β-methylbutyrate supplementation in elderly and clinical populations. Nutrition. (2013) 29:29–36. doi: 10.1016/j.nut.2012.05.005, 23085015

[13] Ologunagba TI Olorundare BO Iyanda T Adewole A Kade IJ. Influence of diphenyl diselenide on thiol redox homeostasis and electrogenic membrane transport in rotenone-induced Parkinson’s disease. bioRxiv. (2022) doi: 10.1101/2022.10.30.514424

[14] Belagodu A Kang S Gulley JM Galvez R. "Effects of β− hydroxy-β-methylbutyrate (HMB) supplementation on biomarkers for cognitive function and electrophysiological processes in aging". In: Editors Martin CR Preedy VR Rajendram R. Factors Affecting Neurological Aging Elsevier (2021). p. 627–36.

[15] Paidi RK Raha S Roy A Pahan K. Muscle-building supplement β-hydroxy β-methylbutyrate binds to PPARα to improve hippocampal functions in mice. Cell Rep. (2023) 42:112717. doi: 10.1016/j.celrep.2023.112717, 37437568 PMC10440158

[16] Yi MC Khosla C. Thiol–disulfide exchange reactions in the mammalian extracellular environment. Annu Rev Chem Biomol Eng. (2016) 7:197–222. doi: 10.1146/annurev-chembioeng-080615-033553, 27023663 PMC4899241

[17] Rosa RM Roesler R Braga AL Saffi J Henriques JA. Pharmacology and toxicology of diphenyl diselenide in several biological models. Braz J Med Biol Res. (2007) 40:1287–304. doi: 10.1590/S0100-879X2006005000171, 18572457

[18] Barcellos AM Abenante L Sarro MT Leo ID Lenardao EJ Perin G . New prospective for redox modulation mediated by organo selenium and organotellurium compounds. Curr Org Chem. (2017) 21:2044–61. doi: 10.2174/1385272820666161020162113

[19] Puntel RL Avila DS Roos DH Pinton S. Mitochondrial effects of organoselenium and organotellurium compounds. Curr Org Chem. (2016) 20:198–210. doi: 10.2174/1385272819666150724234948, 34638861

[20] Obieziurska-Fabisiak M Pacuła-Miszewska AJ Laskowska A Ścianowski J. Organoselenium compounds as antioxidants. ARKIVOC Online J Org Chem. (2023) 2023:69–92. doi: 10.24820/ark.5550190.p011.908

[21] Birmann PT Casaril AM Abenante L Penteado F Brüning CA Savegnago L . Neuropharmacology of organoselenium compounds in mental disorders and degenerative diseases. Curr Med Chem. (2023) 30:2357–95. doi: 10.2174/0929867329666220615124412, 35708081

[22] Hassan W Oliveira CS Noreen H Kamdem JP Nogueira CW Rocha JBT. Organoselenium compounds as potential neuroprotective therapeutic agents. Curr Org Chem. (2016) 20:218–31. doi: 10.2174/1385272819666150810222632, 42085841

[23] Holeček M. Beta-hydroxy-beta-methylbutyrate supplementation and skeletal muscle in healthy and muscle-wasting conditions. J Cachexia Sarcopenia Muscle. (2017) 8:529–41. doi: 10.1002/jcsm.12208, 28493406 PMC5566641

[24] Courel-Ibáñez J Vetrovsky T Dadova K Pallarés JG Steffl M. Health benefits of β-hydroxy-β-methylbutyrate (HMB) supplementation in addition to physical exercise in older adults: a systematic review with meta-analysis. Nutrients. (2019) 11:11. doi: 10.3390/nu11092082, 31484462 PMC6769498

[25] Slater GJ Jenkins D. Beta-hydroxy-beta-methylbutyrate (HMB) supplementation and the promotion of muscle growth and strength. Sports Med. (2000) 30:105–16. doi: 10.2165/00007256-200030020-00004, 10966150

[26] Shanthaveerappa RS. Insulin Resistance in Health and Disease: Exploring the Role of Disuse Skeletal Muscle Atrophy, Testosterone and Beta-Hydroxy-Beta-Methylbutyrate (HMB) in Young and Older Men. Nottingham: University of Nottingham (2021).

[27] Liao Y-H. The Effects of [beta]-Hydroxy-[beta]-Methylbutyrate (HMB) and Leucine on Cellular Signaling Pathways Controlling Protein Synthesis and Degradation During Sedentary and Post-Exercise Recovery in Skeletal Muscle. Austin, Texas: The University of Texas (2012).

[28] Su H Zhou H Gong Y Xiang S Shao W Zhao X . The effects of β-hydroxy-β-methylbutyrate or HMB-rich nutritional supplements on sarcopenia patients: a systematic review and meta-analysis. Front Med. (2024) 11:1348212. doi: 10.3389/fmed.2024.1348212, 39071082 PMC11272589

[29] Vallejo J Spence M Cheng AL Brotto L Edens NK Garvey SM . Cellular and physiological effects of dietary supplementation with β-hydroxy-β-methylbutyrate (HMB) and β-alanine in late middle-aged mice. PLoS One. (2016) 11:e0150066. doi: 10.1371/journal.pone.0150066, 26953693 PMC4783107

[30] Jana M Prieto S Gorai S Dasarathy S Kundu M Pahan K. Muscle-building supplement β-hydroxy β-methylbutyrate stimulates the maturation of oligodendroglial progenitor cells to oligodendrocytes. J Neurochem. (2024) 168:1340–58. doi: 10.1111/jnc.16084, 38419348 PMC11260247

[31] Paidi RK Pahan K. The neuroprotective potentiality of β-hydroxy β-methyl butyrate in an Alzheimer's disease mouse model. Alzheimers Dement. (2025) 21:e098487. doi: 10.1002/alz70859_098487

[32] Kramer AF Erickson KI. Capitalizing on cortical plasticity: influence of physical activity on cognition and brain function. Trends Cogn Sci. (2007) 11:342–8. doi: 10.1016/j.tics.2007.06.009, 17629545

[33] Rossi M. Ageing-Related Impairments at the Neuromuscular Junction: Nutritional and Exercise Interventions to Attenuate Neuromuscular Dysfunctions. Pavia, Italy: Università degli Studi di Pavia (2022).

[34] Polidori MC Mecocci P Cherubini A Senin U. Physical activity and oxidative stress during aging. Int J Sports Med. (2000) 21:154–7. doi: 10.1055/s-2000-8881, 10834344

[35] Meng Q Su CH. The impact of physical exercise on oxidative and nitrosative stress: balancing the benefits and risks. Antioxidants. (2024):13:573. doi: 10.3390/antiox13050573 38790678 PMC11118032

[36] Dutta D Paidi RK Raha S Roy A Chandra S Pahan K. Treadmill exercise reduces α-synuclein spreading via PPARα. Cell Rep. (2022) 40:111058. doi: 10.1016/j.celrep.2022.111058, 35830804 PMC9308946

[37] Rangasamy SB Jana M Dasarathi S Kundu M Pahan K. Treadmill workout activates PPARα in the hippocampus to upregulate ADAM10, decrease plaques and improve cognitive functions in 5XFAD mouse model of Alzheimer’s disease. Brain Behav Immun. (2023) 109:204–18. doi: 10.1016/j.bbi.2023.01.009, 36682514 PMC10023420

[38] Szuhany KL Bugatti M Otto MW. A meta-analytic review of the effects of exercise on brain-derived neurotrophic factor. J Psychiatr Res. (2015) 60:56–64. doi: 10.1016/j.jpsychires.2014.10.003, 25455510 PMC4314337

[39] Gleeson M Bishop NC Stensel DJ Lindley MR Mastana SS Nimmo MA. The anti-inflammatory effects of exercise: mechanisms and implications for the prevention and treatment of disease. Nat Rev Immunol. (2011) 11:607–15. doi: 10.1038/nri3041, 21818123

[40] Yan Z Lira VA Greene NP. Exercise training-induced regulation of mitochondrial quality. Exerc Sport Sci Rev. (2012) 40:159–64. doi: 10.1097/JES.0b013e3182575599, 22732425 PMC3384482

[41] Erickson KI Voss MW Prakash RS Basak C Szabo A Chaddock L . Exercise training increases size of hippocampus and improves memory. Proc Natl Acad Sci USA. (2011) 108:3017–22. doi: 10.1073/pnas.1015950108, 21282661 PMC3041121

[42] Villareal DT Chode S Parimi N Sinacore DR Hilton T Armamento-Villareal R . Weight loss, exercise, or both and physical function in obese older adults. N Engl J Med. (2011) 364:1218–29. doi: 10.1056/NEJMoa1008234, 21449785 PMC3114602

[43] Pedersen BK. Physical activity and muscle-brain crosstalk. Nat Rev Endocrinol. (2019) 15:383–92. doi: 10.1038/s41574-019-0174-x, 30837717

[44] Mugesh G du Mont WW Sies H. Chemistry of biologically important synthetic organoselenium compounds. Chem Rev. (2001) 101:2125–80. doi: 10.1021/cr000426w, 11710243

[45] Quispe RL Jaramillo ML Galant LS Engel D Dafre AL Teixeira da Rocha JB . Diphenyl diselenide protects neuronal cells against oxidative stress and mitochondrial dysfunction: involvement of the glutathione-dependent antioxidant system. Redox Biol. (2019) 20:118–29. doi: 10.1016/j.redox.2018.09.014, 30308475 PMC6176650

[46] Kornasio R Riederer I Butler-Browne G Mouly V Uni Z Halevy O. Beta-hydroxy-beta-methylbutyrate (HMB) stimulates myogenic cell proliferation, differentiation and survival via the MAPK/ERK and PI3K/Akt pathways. Biochim Biophys Acta. (2009) 1793:755–63. doi: 10.1016/j.bbamcr.2008.12.017, 19211028

[47] Baptista IL Silva WJ Artioli GG Guilherme JP Leal ML Aoki MS . Leucine and HMB differentially modulate proteasome system in skeletal muscle under different sarcopenic conditions. PLoS One. (2013) 8:e76752. doi: 10.1371/journal.pone.0076752, 24124592 PMC3790739

[48] Feng L Li B Xi Y Cai M Tian Z. Aerobic exercise and resistance exercise alleviate skeletal muscle atrophy through IGF-1/IGF-1R-PI3K/Akt pathway in mice with myocardial infarction. Am J Physiol Cell Physiol. (2022) 322:C164–76. doi: 10.1152/ajpcell.00344.2021, 34852207

[49] Leal LG Lopes MA Batista ML Jr. Physical exercise-induced Myokines and muscle-adipose tissue crosstalk: a review of current knowledge and the implications for health and metabolic diseases. Front Physiol. (2018) 9:1307. doi: 10.3389/fphys.2018.01307, 30319436 PMC6166321

[50] Pesarico AP Cechella JL Nogueira CW Rosa SG. Swimming exercise and diphenyl diselenide-supplemented diet modulate cerebral cortical and striatal GABA uptake in aged rats. An Acad Bras Cienc. (2022) 94:e20200844. doi: 10.1590/0001-3765202220200844, 35019002

[51] Cechella JL Leite MR Pinton S Zeni G Nogueira CW. Neuroprotective benefits of aerobic exercise and organoselenium dietary supplementation in Hippocampus of old rats. Mol Neurobiol. (2018) 55:3832–40. doi: 10.1007/s12035-017-0600-9, 28540659

[52] Heck SO Fulco BC Quines CB Oliveira CE Leite MR Cechella JL . Combined therapy with swimming exercise and a diet supplemented with diphenyl diselenide is effective against age-related changes in the hepatic metabolism of rats. J Cell Biochem. (2017) 118:1574–82. doi: 10.1002/jcb.25819, 27918086

[53] Leite MR Cechella JL Pinton S Nogueira CW Zeni G. A diphenyl diselenide-supplemented diet and swimming exercise promote neuroprotection, reduced cell apoptosis and glial cell activation in the hypothalamus of old rats. Exp Gerontol. (2016) 82:1–7. doi: 10.1016/j.exger.2016.05.006, 27215802

[54] Cechella JL Leite MR Rosario AR Sampaio TB Zeni G. Diphenyl diselenide-supplemented diet and swimming exercise enhance novel object recognition memory in old rats. Age (Dordr). (2014) 36:9666. doi: 10.1007/s11357-014-9666-8, 24994534 PMC4150883

[55] Leite MR Cechella JL Mantovani AC Duarte MM Nogueira CW Zeni G. Swimming exercise and diphenyl diselenide-supplemented diet affect the serum levels of pro- and anti-inflammatory cytokines differently depending on the age of rats. Cytokine. (2015) 71:119–23. doi: 10.1016/j.cyto.2014.09.006, 25307207

[56] Da Rosa PC Hartmann DD Stefanello ST da Silva TC Leite MT Souza MB . Diphenyl diselenide blunts swimming training on mitochondrial liver redox adaptation mechanisms of aged animals. Sport Sci Health. (2020) 16:281–90. doi: 10.1007/s11332-019-00603-8

[57] Ramos-Hernández R Miguel-Ortega Á Martínez-Ferrán M Fernández-Lázaro D Busto N Mielgo-Ayuso J. Combined creatine and HMB co-supplementation improves functional strength independent of muscle mass in physically active older adults: a randomized crossover trial. Geroscience. (2025). doi: 10.1007/s11357-025-01889-y, 41073834 PMC13356226

[58] Gutiérrez-Reguero H Buendía-Romero Á Franco-López F Martínez-Cava A Hernández-Belmonte A Courel-Ibáñez J . Effects of multicomponent training and HMB supplementation on disability, cognitive and physical function in institutionalized older adults aged over 70 years: a cluster-randomized controlled trial. J Nutr Health Aging. (2024) 28:100208. doi: 10.1016/j.jnha.2024.100208, 38489992 PMC12877245

[59] Meza-Valderrama D Sánchez-Rodríguez D Messaggi-Sartor M Muñoz-Redondo E Morgado-Pérez A Tejero-Sánchez M . Supplementation with β-hydroxy-β-methylbutyrate after resistance training in post-acute care patients with sarcopenia: a randomized, double-blind placebo-controlled trial. Arch Gerontol Geriatr. (2024) 119:105323. doi: 10.1016/j.archger.2023.105323, 38171034

[60] Yang C Song Y Li T Chen X Zhou J Pan Q . Effects of beta-hydroxy-beta-methylbutyrate supplementation on older adults with sarcopenia: a randomized, double-blind, placebo-controlled study. J Nutr Health Aging. (2023) 27:329–39. doi: 10.1007/s12603-023-1911-1, 37248756 PMC12878579

[61] Aoki K Konno M Tokinoya K Honda K Abe T Nagata T . Long-term habitual exercise and combination of β-hydroxy-β-methylbutyrate plus black ginger alter the autophagy and mitochondria related genes in SAMP8 mice. J Nutr Sci Vitaminol. (2022) 68:39–46. doi: 10.3177/jnsv.68.39, 35228494

[62] Rathmacher JA Pitchford LM Khoo P Angus H Lang J Lowry K . Long-term effects of calcium β-hydroxy-β-methylbutyrate and vitamin D3 supplementation on muscular function in older adults with and without resistance training: a randomized, double-blind, controlled study. J Gerontol A Biol Sci Med Sci. (2020) 75:2089–97. doi: 10.1093/gerona/glaa218, 32857128 PMC7566440

[63] Bennett BT Mohamed JS Alway SE. The effects of calcium-β-hydroxy-β-methylbutyrate on aging-associated apoptotic signaling and muscle mass and function in unloaded but nonatrophied extensor digitorum longus muscles of aged rats. Oxidative Med Cell Longev. (2020) 2020:1–18. doi: 10.1155/2020/3938672, 32774671 PMC7396042

[64] Courel-Ibáñez J Pallarés JG. Effects of β-hydroxy-β-methylbutyrate (HMB) supplementation in addition to multicomponent exercise in adults older than 70 years living in nursing homes, a cluster randomized placebo-controlled trial: the HEAL study protocol. BMC Geriatr. (2019) 19:188. doi: 10.1186/s12877-019-1200-5, 31277595 PMC6612176

[65] Din USU Brook MS Selby A Quinlan J Boereboom C Abdulla H . A double-blind placebo controlled trial into the impacts of HMB supplementation and exercise on free-living muscle protein synthesis, muscle mass and function, in older adults. Clin Nutr. (2019) 38:2071–8. doi: 10.1016/j.clnu.2018.09.025, 30360984 PMC6876270

[66] Hobson RM Saunders B Ball G Harris RC Sale C. Effects of β-alanine supplementation on exercise performance: a meta-analysis. Amino Acids. (2012) 43:25–37. doi: 10.1007/s00726-011-1200-z, 22270875 PMC3374095

[67] Saunders B Elliott-Sale K Artioli GG Swinton PA Dolan E Roschel H . Β-Alanine supplementation to improve exercise capacity and performance: a systematic review and meta-analysis. Br J Sports Med. (2017) 51:658–69. doi: 10.1136/bjsports-2016-096396, 27797728

[68] Derave W Everaert I Beeckman S Baguet A. Muscle carnosine metabolism and beta-alanine supplementation in relation to exercise and training. Sports Med. (2010) 40:247–63. doi: 10.2165/11530310-000000000-00000, 20199122

[69] Pence BD Gibbons TE Bhattacharya TK Mach H Ossyra JM Petr G . Effects of exercise and dietary epigallocatechin gallate and β-alanine on skeletal muscle in aged mice. Appl Physiol Nutr Metab. (2016) 41:181–90. doi: 10.1139/apnm-2015-0372, 26761622

[70] Culbertson JY Kreider RB Greenwood M Cooke M. Effects of beta-alanine on muscle carnosine and exercise performance: a review of the current literature. Nutrients. (2010) 2:75–98. doi: 10.3390/nu2010075, 22253993 PMC3257613

[71] Paidi RK Gorai S Mondal S Pahan K. L-leucine upregulates lysosomal biogenesis and autophagy to lower plaques in 5XFAD mouse model of Alzheimer's disease. J Neurochem. (2026) 170:e70432. doi: 10.1111/jnc.70432, 41940752 PMC13070289

[72] Russ DW Acksel C McCorkle KW Edens NK Garvey SM. Effects of running wheel activity and dietary HMB and β-alanine co-supplementation on muscle quality in aged male rats. J Nutr Health Aging. (2017) 21:554–61. doi: 10.1007/s12603-016-0810-2, 28448086 PMC12878543

[73] Stout JR Fukuda DH Kendall KL Smith-Ryan AE Moon JR Hoffman JR. Β-Hydroxy-β-methylbutyrate (HMB) supplementation and resistance exercise significantly reduce abdominal adiposity in healthy elderly men. Exp Gerontol. (2015) 64:33–4. doi: 10.1016/j.exger.2015.02.012, 25700845

[74] Stout JR Smith-Ryan AE Fukuda DH Kendall KL Moon JR Hoffman JR . Effect of calcium β-hydroxy-β-methylbutyrate (CaHMB) with and without resistance training in men and women 65+yrs: a randomized, double-blind pilot trial. Exp Gerontol. (2013) 48:1303–10. doi: 10.1016/j.exger.2013.08.007, 23981904

[75] Kim JS Park YM Lee SR Masad IS Khamoui AV Jo E . Β-hydroxy-β-methylbutyrate did not enhance high intensity resistance training-induced improvements in myofiber dimensions and myogenic capacity in aged female rats. Mol Cells. (2012) 34:439–48. doi: 10.1007/s10059-012-0196-x, 23149873 PMC3887788

[76] Hao Y Jackson JR Wang Y Edens N Pereira SL Alway SE. Β-Hydroxy-β-methylbutyrate reduces myonuclear apoptosis during recovery from hind limb suspension-induced muscle fiber atrophy in aged rats. Am J Physiol Regul Integr Comp Physiol. (2011) 301:R701–15. doi: 10.1152/ajpregu.00840.2010, 21697520 PMC3174754

[77] Osuka Y Kojima N Sasai H Wakaba K Miyauchi D Tanaka K . Effects of exercise and/or β-hydroxy-β-methylbutyrate supplementation on muscle mass, muscle strength, and physical performance in older women with low muscle mass: a randomized, double-blind, placebo-controlled trial. Am J Clin Nutr. (2021) 114:1371–85. doi: 10.1093/ajcn/nqab176, 34081113

[78] Flakoll P Sharp R Baier S Levenhagen D Carr C Nissen S. Effect of beta-hydroxy-beta-methylbutyrate, arginine, and lysine supplementation on strength, functionality, body composition, and protein metabolism in elderly women. Nutrition. (2004) 20:445–51. doi: 10.1016/j.nut.2004.01.009, 15105032

[79] Din USU. Application of Novel Deuterium Oxide Tracer Techniques to Determine the Effects of ß-Hydroxy ß-Methylbutyrate (HMB) Supplementation and Exercise in Ageing: a Randomized Placebo Controlled Trial. Nottingham: University of Nottingham. (2016).

[80] Rondanelli M Gasparri C Cavioni A Sivieri C Barrile GC Mansueto F . A patented dietary supplement (hydroxy-methyl-butyrate, carnosine, magnesium, butyrate, lactoferrin) is a promising therapeutic target for age-related sarcopenia through the regulation of gut permeability: a randomized controlled trial. Nutrients. (2024) 16:1369. doi: 10.3390/nu16091369, 38732615 PMC11085744

[81] Berton L Bano G Carraro S Veronese N Pizzato S Bolzetta F . Effect of oral beta-hydroxy-beta-methylbutyrate (HMB) supplementation on physical performance in healthy old women over 65 years: an open label randomized controlled trial. PLoS One. (2015) 10:e0141757. doi: 10.1371/journal.pone.0141757, 26529601 PMC4631374

[82] Park Y-M. Effects of Beta-Hydroxy Βeta-Methylbutyrate (HMB) on Skeletal Muscles of Aged Sprague-Dawley Female Rats during 10-Week Intensive Resistance Exercise Training, vol. 24 Tallahassee, Florida: Florida State University (2010).

